# Combined Transcriptomic and Proteomic Profiling of *E. coli* under Microaerobic versus Aerobic Conditions: The Multifaceted Roles of Noncoding Small RNAs and Oxygen-Dependent Sensing in Global Gene Expression Control

**DOI:** 10.3390/ijms23052570

**Published:** 2022-02-25

**Authors:** Gunn-Guang Liou, Anna Chao Kaberdina, Wei-Syuan Wang, Vladimir R. Kaberdin, Sue Lin-Chao

**Affiliations:** 1 Institute of Molecular Biology, Academia Sinica, Taipei 11529, Taiwan; bogun@gate.sinica.edu.tw (G.-G.L.); 2010kaberdina@gmail.com (A.C.K.); wesswang@gmail.com (W.-S.W.); 2 Molecular and Cell Biology, Taiwan International Graduate Program, Institute of Molecular Biology, Academia Sinica and Graduate Institute of Life Sciences, National Defense Medical Center, Taipei 11490, Taiwan; 3 Department of Immunology, Microbiology and Parasitology, University of the Basque Country UPV/EHU, 48940 Leioa, Spain; 4 Basque Foundation for Science, IKERBASQUE, Maria Diaz de Haro 3, 48013 Bilbao, Spain; 5 Research Centre for Experimental Marine Biology and Biotechnology (PIE-UPV/EHU), 48620 Plentzia, Spain

**Keywords:** transcriptional and post-transcriptional regulation, iron homeostasis, anaerobic respiration, acid stress response

## Abstract

Adaptive mechanisms that facilitate intestinal colonization by the human microbiota, including *Escherichia coli*, may be better understood by analyzing the physiology and gene expression of bacteria in low-oxygen environments. We used high-throughput transcriptomics and proteomics to compare the expression profiles of *E. coli* grown under aerobic versus microaerobic conditions. Clustering of high-abundance transcripts under microaerobiosis highlighted genes controlling acid-stress adaptation (*gadAXW*, *gadAB*, *hdeAB-yhiD* and *hdeD* operons), cell adhesion/biofilm formation (*pgaABCD* and *csgDEFG* operons), electron transport (*cydAB*), oligopeptide transport (*oppABCDF*), and anaerobic respiration/fermentation (*hyaABCDEF* and *hycABCDEFGHI* operons). In contrast, downregulated genes were involved in iron transport (*fhuABCD*, *feoABC* and *fepA-entD* operons), iron-sulfur cluster assembly (*iscRSUA* and *sufABCDSE* operons), aerobic respiration (*sdhDAB* and *sucABCDSE* operons), and de novo nucleotide synthesis (*nrdHIEF*). Additionally, quantitative proteomics showed that the products (proteins) of these high- or low-abundance transcripts were expressed consistently. Our findings highlight interrelationships among energy production, carbon metabolism, and iron homeostasis. Moreover, we have identified and validated a subset of differentially expressed noncoding small RNAs (i.e., CsrC, RyhB, RprA and GcvB), and we discuss their regulatory functions during microaerobic growth. Collectively, we reveal key changes in gene expression at the transcriptional and post-transcriptional levels that sustain *E. coli* growth when oxygen levels are low.

## 1. Introduction

*Escherichia coli* is a Gram-negative commensal bacterium that commonly inhabits the intestines of humans and other animals under microaerobic or anaerobic conditions. Previous studies have shown that *E. coli* growth at different concentrations of oxygen involves substantial reprogramming of the gene expression controlled by several transcription factors, such as ArcA and FNR, which together enable *E. coli* to adapt to and survive under altered oxygen availabilities [1,2,3,4,5,6,7]. ArcA belongs to the two-component ArcAB system, which functions as a microaerobic redox regulator, whereas FNR is known for its major regulatory role in the transition from aerobic to anaerobic growth through the activation of genes involved in anaerobic metabolism and repression of genes involved in aerobic metabolism. Previous studies have revealed that during the switch from anaerobiosis to aerobiosis and vice versa, *E. coli* reprograms gene expression to adjust its cellular metabolism, energy production, and iron homeostasis. Recently, several studies have analyzed the impact of oxygen availability on genome-wide expression profiles, focusing on specific regulatory systems or changes associated with aerobic–anaerobic transitions in *E. coli* [6,7,8,9,10,11,12,13,14,15,16].

Despite some progress, our understanding of microaerobic/anaerobic growth and adaptation remains limited due to the lack of an integrated overview of the transcriptional and post-transcriptional events involved in these processes. These missing links in the regulatory network are particularly critical for gaining in-depth insights into the mechanisms and gene expression patterns that define the physiology, reproduction, and growth of bacteria (both commensal and pathogenic) in the intestine and other deep tissues.

These knowledge gaps prompted us to conduct concomitant transcriptomic and proteomic analyses of both microaerobic and aerobic *E. coli* cultures to identify the genes and their products that play key roles in adaptation to low oxygen concentrations. Our results reveal major gene clusters essential for sustaining microaerobic growth and uncover how they may be regulated by several noncoding small regulatory RNAs (sRNAs), including RyhB, RprA, GcvB, CsrB and CsrC.

## 2. Results

### 2.1. Generation and Quality Assessment of Aerobic and Microaerobic Transcriptomes of E. coli Cultures Grown on Defined Media

*E. coli* MG1655 strain was grown on minimal medium containing glucose as a carbon source under continuous aerobic or microaerobic conditions. These experiments were carried out in a benchtop fermentor (Winpact Parallel Fermentation System FS-05-220) to obtain multiple biological replicates (namely, biological replicates of 5 aerobic and 10 microaerobic cultures) for further analysis. Cell doubling times of aerobic and microaerobic cultures were 77.9 ± 8.6 and 245.5 ± 24.7 min, respectively. To prepare total RNA [17] or protein samples, cells were grown to mid-logarithmic phase, corresponding to OD_460_ 0.5–0.6. Samples of purified RNA were used for the RNA deep-sequencing analyses, and the same batches of RNA were used for Northern blot validation. Details of the bioreactor culture conditions, RNA deep-sequencing procedures, and reagents used are provided in the Materials and Methods.

More than 9 million raw sequencing reads were generated for each RNA sample. After trimming the raw sequencing reads, the high-quality unique sequences were mapped to the *E. coli* K12 substrain MG1655 reference genome (NC_000913) [18]. On average, mapping coverages of 94.6% and 88.8% were obtained for total unique sequence reads under aerobic and microaerobic growth conditions, respectively (Table S1). With a threshold of ≥1 transcript per million mapped reads (TPM), we detected expression of 4388 and 4385 genes under aerobic and microaerobic growth conditions, respectively (NCBI GEO accession # GSE189154).

To assess the robustness of our datasets, we calculated correlations across biological replicates. In Figure S1A, we show values for the correlation coefficients for all pairwise scatterplots obtained for aerobic or microaerobic samples. We identified strong correlations between biological replicates representing the same growth condition. More specifically, correlation coefficients for aerobic and microaerobic transcriptomes were greater than 0.97 and 0.91, respectively, implying that our results are highly reproducible. In addition, we performed principal component analysis (PCA) on a combined dataset that successively maximized variance across all datasets (i.e., datasets O-1~O-5 and N-1~N-10). Our PCA revealed two distinct groups (i.e., O-1~O-5 and N-1~N-10) corresponding to aerobic and microaerobic cultures, respectively (Figure S1B). Together, our correlation and PCA analyses justify the use of all our RNA-seq datasets for further gene expression analyses.

### 2.2. Defining Major Gene Clusters Involved in Adaptation to Microaerobiosis

To identify differentially expressed genes (DEGs), we ranked the processed data on detected gene transcripts under aerobic (4388 genes) and microaerobic (4385 genes) conditions according to false discovery rate (FDR)-adjusted *p*-values and log_2_ fold-change (FC).

In order to group DEGs according to their biological functions, the DEGs were functionally annotated using various bioinformatics tools—linked to STRING [19,20,21], gene ontology (GO) [22,23,24], Kyoto Encyclopedia of Genes and Genomes (KEGG) pathway [25,26,27], UniProtKB keyword [28,29,30,31], RegulonDB [32], and EcoCyc [2,33] databases—and were then clustered according to those annotations. Moreover, in order to determine the specific contributions of regulatory factors, we also analyzed the gene expression levels of transcription factors (see Section 2.2.4), prophage-related genes (see Section 2.2.3), and small regulatory RNAs (see Section 2.3).

#### 2.2.1. Identification of DEGs under Microaerobic Versus Aerobic Conditions

**Figure 1:**
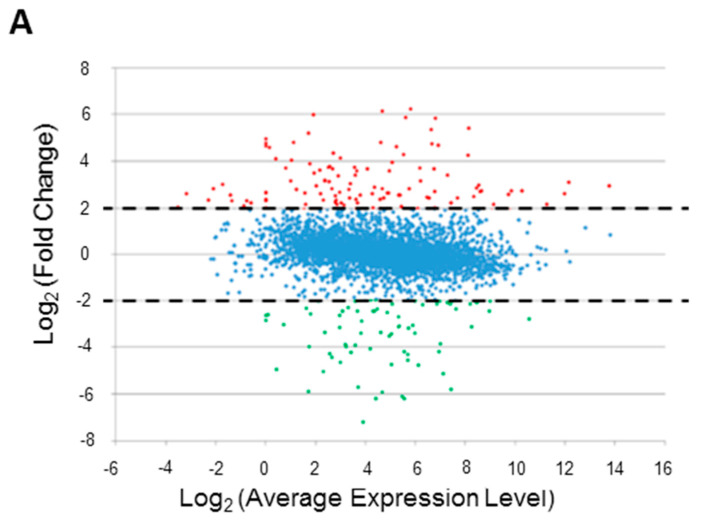

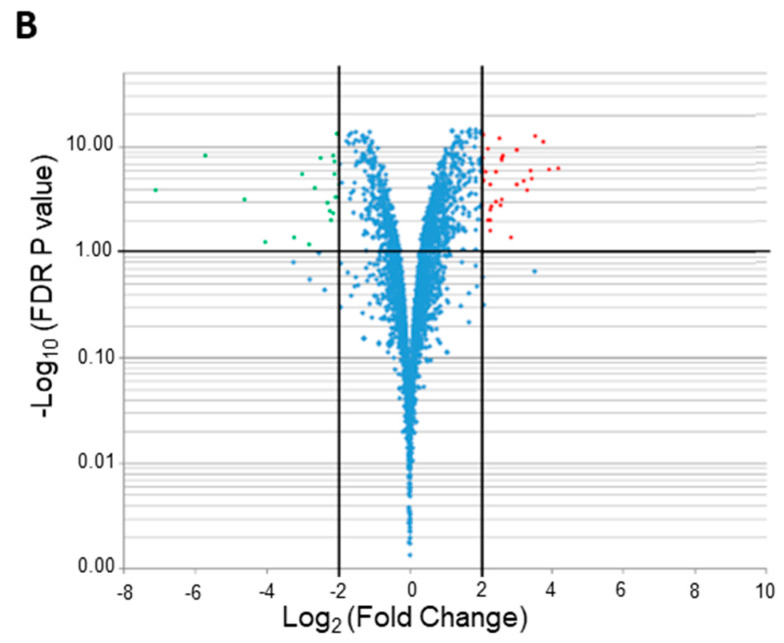
Identification of differentially expressed genes (DEGs). (**A**) MA plot showing the relationship between gene expression level (A values on the x axis) and fold-change (FC) (M values on the y axis) across genes. The discontinuous horizontal black lines indicate the fold-change (FC) threshold applied (absolute value of log_2_ FC ≥ 2). DEGs displaying statistical significance (i.e., meeting this FC criterion) are shown as red (176 upregulated genes) or green (104 downregulated genes) dots. (**B**) Volcano plot displaying FC plotted against the false discovery rate (FDR) *p*-value. The y axis represents the −log_10_ FDR *p*-value and the x axis represents the log_2_ FC value. The horizontal black line indicates the significance threshold (−log_10_ *p*-value ≥ 1), and the vertical black lines indicate the FC threshold (absolute value of log_2_ FC ≥ 2). DEGs displaying statistical significance (i.e., those meeting both criteria) are shown as 105 upregulated (red dots) and 71 downregulated (green dots) genes in the right-upper and left-upper areas of the panel delineated by black lines, respectively.

To compare the gene expression patterns of microaerobic and aerobic cultures, we calculated the normalized transcript expression values (expressed as TPM) for each gene with a *p*-value ≤ 0.05 and selected 280 DEG transcripts displaying log_2_ fold-change ≥ 2, representing 176 upregulated and 104 downregulated genes, respectively (Figure 1A). Next, we filtered these DEGs using a FDR threshold of −log_10_ (*p*-value) ≥ 1 (Figure 1B), which resulted in 176 DEGs (105 upregulated and 71 downregulated genes) that showed significant differential expression.

#### 2.2.2. Functional Clusters of DEGs

**Figure 2:**
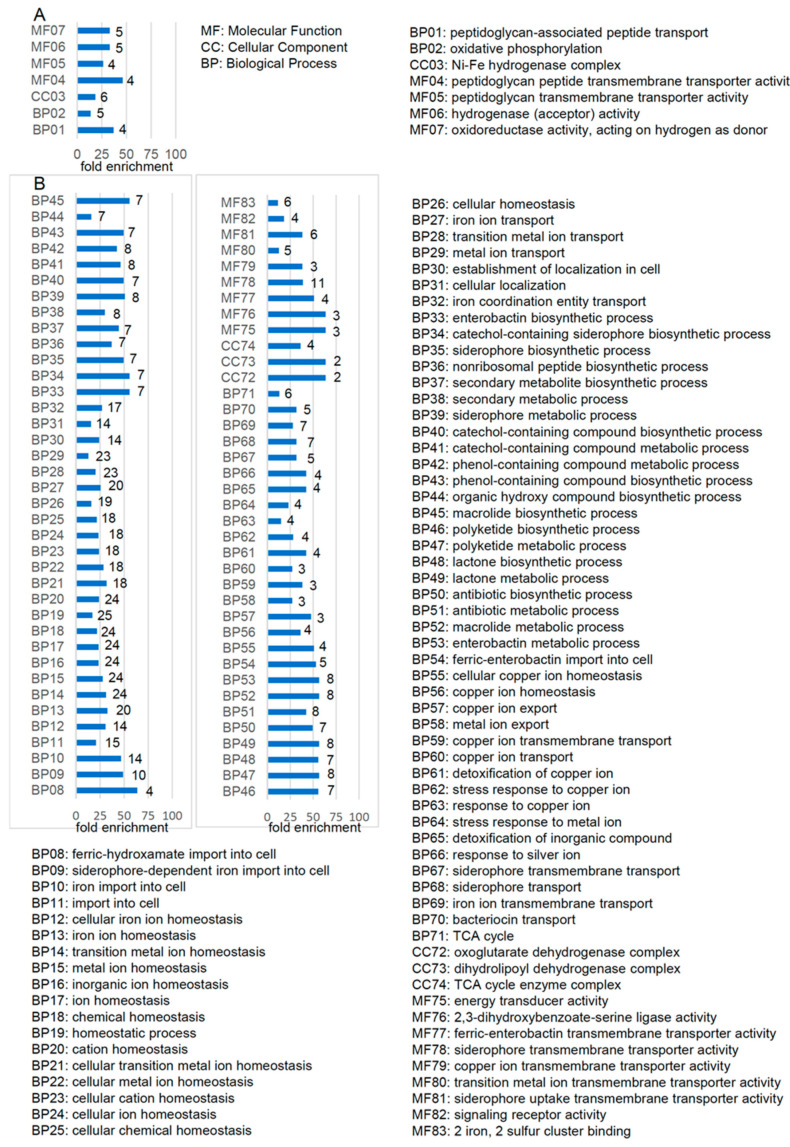

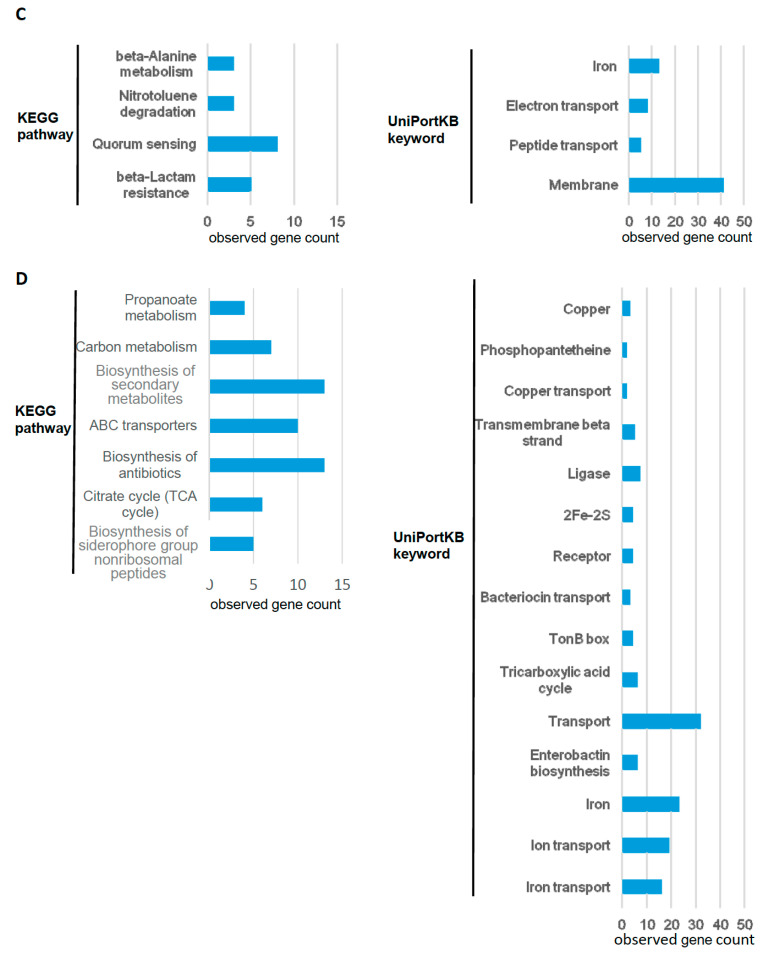
Functional classification of the differentially expressed genes (DEGs). (**A**,**B**) Gene ontology (GO) enrichment terms were categorized into biological process (BP), cellular component (CC), or molecular function (MF) for upregulated DEGs (**A**) and downregulated DEGs (**B**). Symbol codes for enriched subcategory terms are shown on the y axis, and fold enrichment is presented on the x axis of the horizontal histogram. Numbers of genes for each enriched subcategory are shown to the right of the respective horizontal bar in the histogram. A list of enriched subcategory terms is shown. (**C**,**D**) Kyoto Encyclopedia of Genes and Genomes (KEGG) pathway enrichment (left panels) and UniProtKB keyword (right panels) analyses were conducted to further classify upregulated (**C**) and downregulated (**D**) DEGs. The enriched term and the respective number of observed genes is shown to the left or right of the histogram, respectively.

We present a comprehensive overview of the identified DEGs in Table 1, revealing key gene clusters, genes/operons, biological functions, small regulatory RNAs, and related transcription factors involved in adaptation to changing oxygen conditions. Upregulated (Figure 2A) and downregulated DEGs (Figure 2B) were functionally classified into three general gene ontology (GO) categories, i.e., biological processes, cellular components, and molecular functions. Based on significant fold enrichment (≥10 compared with reference genes in the same subcategory), the upregulated DEGs we identified were mainly found in the following GO subcategories: peptidoglycan-associated peptide transport, oxidative phosphorylation, Ni-Fe hydrogenase complex, peptidoglycan peptide transmembrane transporter activity, peptidoglycan transmembrane transporter activity, hydrogenase (acceptor) activity, and oxidoreductase activity acting on hydrogen as donor (Figure 2A). Through the same process, we identified 76 GO subcategories for downregulated DEGs, including TCA cycle, several transport processes (such as ferric hydroxamate import into cell, iron import into cell, copper ion export), ion homeostasis, chemical homeostasis, enterobactin biosynthetic process, nonribosomal peptide biosynthetic process, secondary metabolite biosynthetic process, lactone metabolic process, antibiotic metabolic process, stress response to metal ion, detoxification of inorganic compound, energy transducer activity, signaling receptor activity, and Fe_2_S_2_ cluster binding, among others (Figure 2B). To better define the molecular interactions, reactions, and relationship network of the biogenesis pathways that are differentially affected under microaerobic versus aerobic conditions, we conducted a Kyoto Encyclopedia of Genes and Genomes (KEGG) pathway enrichment analysis (*p*-value ≤ 0.05) on the DEGs. This analysis revealed four and seven KEGG pathways that were upregulated or downregulated, respectively (Figure 2C,D, left panels, respectively). The upregulated KEGG pathways were β-alanine metabolism, nitrotoluene degradation, quorum sensing, and β-lactam resistance, whereas the downregulated KEGG pathways were propanoate metabolism, carbon metabolism, biosynthesis of secondary metabolites, ABC transporters, biosynthesis of antibiotics, TCA cycle, and biosynthesis of siderophore group nonribosomal peptides.

We also employed the UniProt Knowledgebase (UniProtKB) keyword database (EMBL-EBI, Cambridge, UK; SIB Swiss Institute of Bioinformatics, Geneva, Switzerland; PIR, Washington, DC, USA) to further characterize the retrieved pathways pertaining to DEGs. By assigning DEGs to functional, structural, or other UniProtKB keyword categories, we essentially generated a highly similar classification to those determined from our GO and KEGG pathway analyses. More specifically, UniProtKB keywords associated with upregulated DEGs were iron, electron transport, peptide transport, and membrane, whereas copper, phosphopantetheine, copper transport, transmembrane beta strand, ligase, Fe_2_S_2_, receptor, bacteriocin transport, TonB box, TCA cycle, transport, enterobactin biosynthesis, iron, ion transport, and iron transport were all associated with downregulated DEGs (Figure 2C,D, right panels, respectively).

#### 2.2.3. Prophage- and Phage-Related Genes

Prophage-related genes constitute up to 13.5% of the *E. coli* genome [34,35], contributing to bacterial survival in hosts by increasing cell fitness and virulence. Recent studies have revealed that the expression of such prophage-related genes can (i) increase *E. coli* resistance to adverse conditions such as exposure to antibiotic, acid, oxidative, or osmotic stress; and (ii) influence metabolic remodeling, biofilm formation, cell movement, and growth [36,37,38,39,40]. Although our recent works revealed that one such prophage gene, *dicF* [41], plays a critical role in regulating cell division under anaerobic conditions [42], how other prophage-related genes are expressed and function under oxygen-limited conditions remains unclear.

To identify other prophage-related genes that potentially contribute to cell fitness and survival in microaerobic environments, we compared the expression of prophage- and phage-related genes under microaerobic versus aerobic conditions. Using our RNA-seq transcriptomic dataset and based on 245 known prophage-related genes within 134 operons [2,33], we detected the expression of 200 prophage-related genes within 118 operons, reflecting 132 upregulated and 68 downregulated genes. As summarized in Table 2, highly upregulated (log_2_ FC ≥ 1.5) prophage- and phage-related genes could be assigned to 15 operons, whereas the downregulated ones were solely localized in the *fhuACDB* operon that codes for proteins involved in ferrichrome transport. Notably, FhuA protein can also serve as a phage receptor [43].

#### 2.2.4. Transcription Factor Genes

Transcription factors (TFs) encompass a large number of regulatory proteins that control gene expression by affecting transcription rates in either a positive (activator) or a negative (repressor) manner. Given their key role in reprogramming gene expression, we compared transcript abundances of 139 TFs [2,33] under microaerobic and aerobic conditions and identified 12 transcripts displaying >2-fold differential expression, including 7 upregulated (*dps*, *evgA, gadW, gadX, leuO*, *pdhR*, and *putA)* and 5 downregulated TF genes (*cusR, fis, fecl, IscR*, and *soxS*) (Table 3).

Interestingly, some of these transcripts encode TFs that control metal ion homeostasis; for instance, CusR for copper, and LscR, Dps, and FecI for iron. Theoretically, when the level of a TF increases, its target genes should be controlled accordingly, depending on whether the TF is a positive or negative regulator. We selected the IscR regulon for further analysis. Levels of the *iscR* mRNA decreased ~4.4-fold (log_2_ = 2.13; Table 3) under microaerobic conditions relative to those under aerobic growth, and the level of the respective protein, lscR, consequently also diminished (~1.3-fold; Table S2). This reduction in IscR abundance, along with an anticipated reduction in [Fe_2_S_2_] iron-sulfur cluster availability (Table 1) required for IscR activity, could be accountable, at least in part, for the nearly 3-fold upregulation of *torT* (log_2_ = 1.67; Table 4). The changes in IscR abundance had the most pronounced effect on the *nrdHIEF* operon, the genes of which exhibited a 10.8–59.3 fold-change (log_2_ = 3.43 to log_2_ = 5.89 in Table 4) in downregulated DEG expression.

Low or high oxygen concentrations greatly impact the redox state and activity of key TFs (e.g., Fur, FNR, ArcA), contributing to differences between aerobic and microaerobic growth [2,5,6,7,8,9,10,32,33,44]. Ferric uptake regulator (Fur) regulates iron metabolism in an iron-dependent manner. As low oxygen conditions apparently increase the concentration of free Fe^2+^ ions, Fe^2+^ binding to Fur increases the concentration of its active form (Fur-Fe^2+^), thereby affecting Fur-dependent gene expression. Indeed, searching for DEGs within the 65 known operons regulated by Fur-Fe^2+^ [2,32,33] (Table 5) revealed that Fur potentially affects the expression of many genes, including those involved in transport of iron-siderophore/ferrichrome complexes (*exbDB*, ~18-fold; log_2_ = 4.2), enterobactin biosynthesis (*entCEBAH*, ~14–~148-fold; log_2_ = 3.9 − 7.2), and regulation of ribonucleotide reductase activity (*nrdHIEF* ~11–~59-fold; log_2_ = 3.4 − 5.9).

The RyhB sRNA acts as a global regulator of iron homeostasis [10]. We observed extremely low abundance of this sRNA under microaerobic conditions (~8-fold less compared to levels under aerobic conditions; log_2_ = 3, Table 5) and it was not detectable by Northern blotting (see Result Section 2.3), an outcome consistent with Fur-dependent repression of *ryhB* transcription.

### 2.3. Identification of Differentially Expressed sRNAs and Northern-Blot-Based Validation

**Figure 3:**
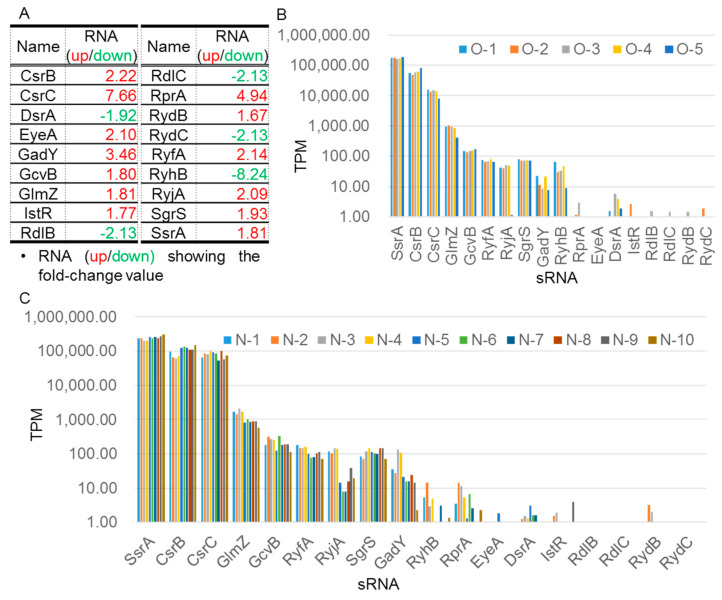
Higher fold-change expression and individual TPM values for known *E. coli* sRNAs detected under microaerobic versus aerobic conditions. (**A**) The fold-change values for upregulated and downregulated sRNAs are shown in red and green, respectively. (**B**,**C**) The y axis shows logarithmic TPM expression values for sRNAs expressed in aerobic (**B**) and microaerobic (**C**) cultures, respectively. The x axis shows the sRNAs listed in (**A**). The individually colored bars represent different biological replicates as indicated.

**Figure 4:**
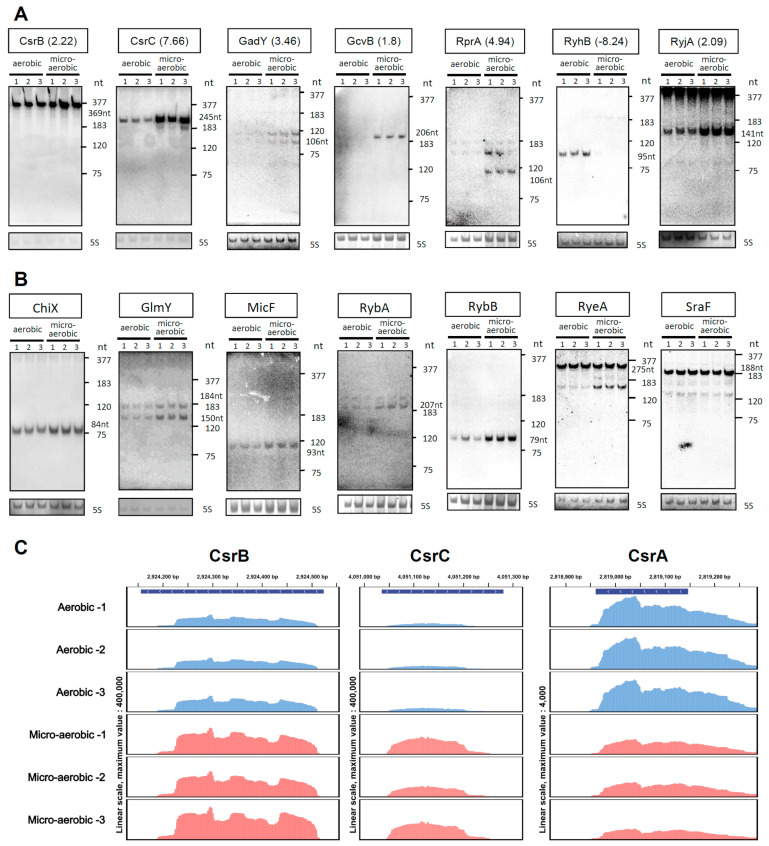
Validation of sRNA expression via Northern blot analysis. (**A**,**B**) Hybridizations were performed with the probes specific for selected sRNAs that showed ≥1.5 fold-change (**A**) or <1.5 fold-change (**B**) in abundance according to our RNA-seq data collected under microaerobic versus aerobic growth conditions. The 5S rRNA served as an internal loading control. The expected sizes (in nucleotides (nt)) of the full-length sRNAs are indicated. The molecular ladder was obtained by hybridizing total RNA with radiolabeled probes specific for RnpB (M1) RNA (377 nt), 6S RNA (183 nt), 5S rRNA (120 nt), and tRNA^Asn^ (75 nt). Three biological replicates were performed and representative images are shown. (**C**) Comparison of reads corresponding to the mapped sRNAs CsrB/C (left and middle panels, respectively) and *csrA* (right panel) mRNAs within the *E. coli* genome. The y axis represents the number of RNA-seq reads for the sRNAs and *csrA* mRNA on the largest scale of 400,000 and 4000, respectively. The coding region of each gene is shown in blue at the top of each panel, and expression is shown in blue and red for aerobic and microaerobic growth conditions, respectively.

**Figure 5:**
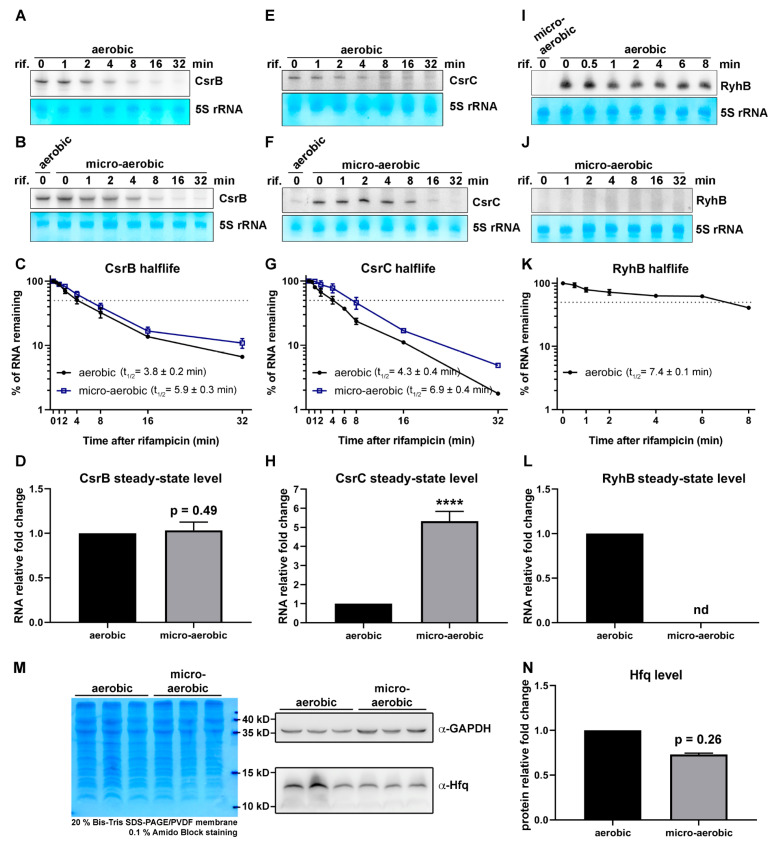
Half-lives of sRNAs CsrB, CsrC, and RyhB and protein abundance of Hfq under aerobic and microaerobic conditions. (**A**–**L**) Northern blot analysis was used to determine the half-lives of CsrB, CsrC, and RyhB under aerobic (**A**, **E**, and **I**, respectively) and microaerobic ((**B**,**F**,**J**), respectively) conditions. Mean values for CsrB (**C**), CsrC (**G**), and RyhB (**K**) half-lives under aerobic and microaerobic conditions are shown (encompassing three biological repeats, bars represent standard error). The dotted gray line indicates 50% of total RNA remaining. Black circles and blue squares represent the signal intensities corresponding to RNA samples from aerobic and microaerobic cultures, respectively. CsrB, CsrC, and RyhB half-lives under aerobic conditions were calculated as 3.8 ± 0.2, 4.3 ± 0.4, and 7.4 ± 0.1 min (**C**,**G**,**K**), respectively, whereas under microaerobic conditions they were 5.5 ± 0.3 min, 6.1 ± 0.4 min, and no detectable signal (see panels (**B**,**F**,**J**)), respectively. Bar graph shows the relative steady-state levels of small RNAs (time 0) normalized to their levels under aerobic conditions, which were arbitrarily set as 1. Experiments were performed with three biological replicates and representative images are shown. The steady-state level of CsrB under microaerobiosis relative to aerobiosis was 1.03 ± 0.05-fold (*p*-value = 0.49) (**D**), whereas for CsrC it was 5.32 ± 0.51-fold (*p*-value < 0.0001, indicated as ****) (**H**). Expression of RyhB was not detectable (nd) under microaerobic conditions (**L**). (**M**) Hfq protein abundance analyzed via Western blotting. Equal amounts of total protein were fractionated in 20% SDS polyacrylamide gels and transferred to a membrane, and the lower part of the membrane was probed with anti-Hfq antibody. The upper part of the membrane was used to detect GAPDH as a loading control. Experiments were performed with three biological replicates and representative images are shown. (**N**) Quantification of Hfq level. The signal obtained with anti-Hfq antibody was normalized using GAPDH and further processed to calculate the relative protein expression level, plotted as vertical bars. Hfq level under microaerobiosis was normalized to its level under aerobiosis, which was arbitrarily set as 1. The difference in Hfq level under these conditions was not statistically significant (*p*-value = 0.26).

sRNAs are common in bacteria, where they play critical roles in regulating a wide range of cellular functions [45]. Our RNA-seq dataset also revealed differential expression of a number of sRNAs under microaerobic and anaerobic conditions. Of the 64 known sRNAs in *E. coli* [2,33], 18 exhibited >1.5-fold difference in abundance under microaerobic versus aerobic conditions (Figure 3A). We employed Northern blot analysis to validate these results. Consistently, we observed that the abundance of RyhB was dramatically reduced under oxygen-limited conditions, rendering it almost undetectable under microaerobiosis (Figure 4A, second panel from right). In contrast, the levels of several other sRNAs (e.g., CsrC, GcvB and RprA) were considerably higher under microaerobiosis (Figure 4A). We detected two or more species of some sRNAs (e.g., GadY and RprA (Figure 4A), GlmY, and RyeA (Figure 4B)). To test whether the increase/decrease in abundance of certain sRNAs could be attributable to their higher metabolic stability, we also determined the half-lives of three sRNAs—namely CsrB, CsrC, and RyhB—by inhibiting their transcription by means of rifampicin treatment, and then determined their time-dependent decreased abundance using Northern blot analysis. As shown in Figure 4A–C,E–G, the half-lives of both CsrC and CsrB increased moderately under microaerobiosis (from 4.3 to 6.9 min and from 3.8 to 5.9 min, respectively), though only the steady-state level of CsrC increased dramatically (Figure 5H). This outcome indicates that it is more efficient transcription, rather than increased RNA stability, that is responsible for the higher CsrC abundance under microaerobic conditions. Given that RyhB was almost undetectable in cells cultured under oxygen-limited conditions, we could not directly compare the half-life of this sRNA under different conditions (Figure 5I–L). In addition to validating sRNA levels by Northern blotting, we also assessed levels of Hfq [46]—an RNA chaperone that plays an important role in facilitating sRNA/mRNA interactions—by means of Western blotting, which revealed only minor differences in abundance under aerobic and microaerobic conditions (Figure 5M,N).

### 2.4. Proteome Analysis Corroborates Differential Protein Abundance under Changing Oxygen Availability

To further validate our DEG analyses, we adopted a quantitative proteomic approach to analyze differential protein abundance under aerobic and microaerobic growth conditions. We conducted this analysis on aliquots of the same batches of cultured cells used for our above-described RNA-seq assays, encompassing two biological repeats for both growth conditions (samples O-1 and O-2 for aerobiosis; samples N-2 and N-3 for microaerobiosis).

#### 2.4.1. Identification of Differentially Abundant Proteins under Microaerobic Versus Aerobic Conditions

We deployed commercially available isobaric iTRAQ * mass tags [47,48] to simultaneously analyze multiple biological samples. The identical masses and chemical properties of these isobaric tags enabled co-elution of various isotopologues. The isobaric tags of peptides were cleaved by collision-induced dissociation (CID) during tandem mass spectrometry (MS/MS), before assessment of peptide fragment ions to define their sequence and quantitation of the isobaric tags. Peptide identification and relative quantitation were determined concurrently. In total, we identified 1498 and 1488 proteins from cells grown under aerobic or microaerobic conditions, respectively (Table S2). Pairwise scatterplots revealed strong correlations between two biological repeats for the same growth condition (r = 0.98 and 0.97 for aerobic and microaerobic growth, respectively) (Figure S2).

We used protein abundance ratios to identify proteins that were differentially abundant under microaerobic growth conditions relative to aerobic growth. Using 95% confidence intervals with two standard deviations (SD), we assumed that ratios >1.39 or <0.698 (i.e., ratios with more than 1.39-fold increase or more than 1.43-fold decrease in protein abundance) indicated significant changes. Accordingly, we set the protein abundance ratio threshold to 1.5 and found 113 and 92 proteins that had increased and decreased in abundance, respectively, in the *E. coli* MG1655 cells grown under microaerobic versus aerobic conditions (Table S3). We explore the consistency among our proteomic and RNA-seq datasets in the Discussion.

#### 2.4.2. Clusters of Proteins with Increased and Decreased Abundance

**Figure 6:**
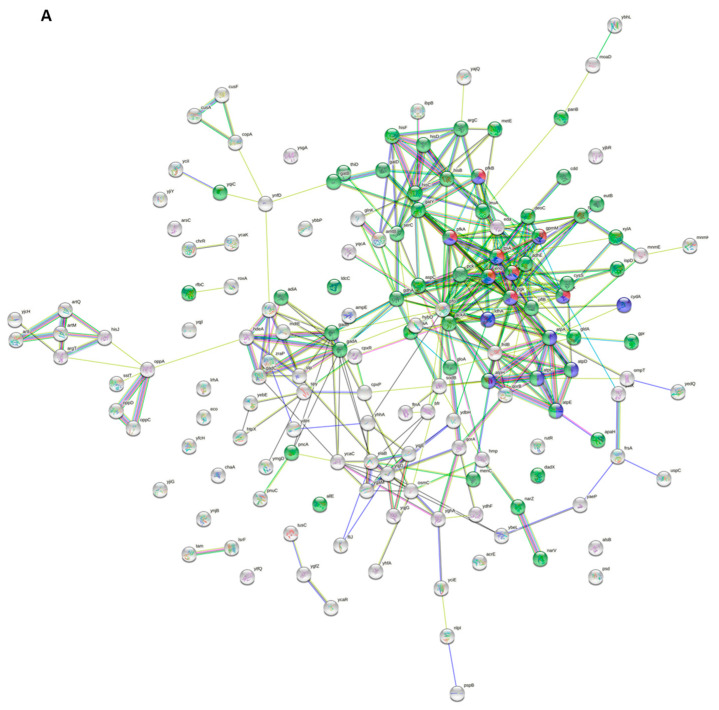

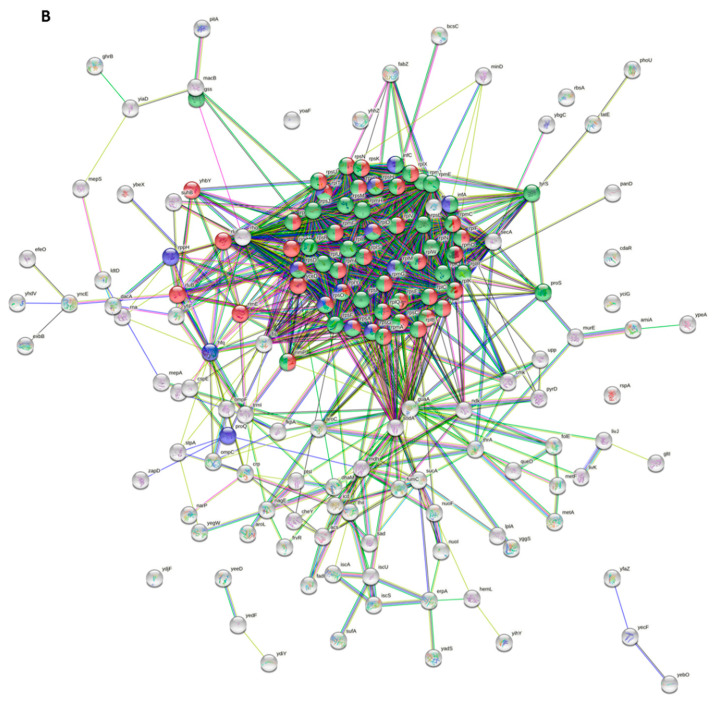
Protein–protein interaction networks of differentially abundant proteins. (**A**,**B**) The protein–protein interaction networks for increased (**A**) and decreased (**B**) differentially abundant proteins were generated using the STRING platform (https://string-db.org/). Abundance-increased proteins were involved in processes such as glycolysis, ATP metabolism, and coenzyme/small-molecule metabolism, for which proteins are represented in red, blue, and green, respectively. Abundance-decreased proteins were involved in ribosome biogenesis, post-transcriptional regulation of gene expression and peptide metabolism, and are indicated in red, blue, and green, respectively.

We applied STRING analysis to determine the protein–protein interaction network of differentially abundant proteins. Furthermore, we also used the EcoCyc [2,33] and RegulonDB [34] databases to highlight translational regulators of these proteins (Table 6). Interestingly, we found that proteins with increased abundance were mainly involved in cellular processes such as glycolysis (e.g., Eno, Glk, GpmI, TpiA, and YdbK), ATP metabolism (e.g., AtpC, AtpD, AtpH, CydA, and LdhA), glucose metabolism (e.g., FbaA, PckA, PflB, SdaA, and TpiA), purine-containing compound metabolism (e.g., AtpA, AtpE, PfkA, PfkB, and YlbA), and small-molecule metabolism such as those responsible for ion homeostasis (e.g., AdiA, CopA, CusF, FtnA, and GadB) (Figure 6A; Table 6). In contrast, proteins that showed decreased abundance were mainly involved in ribosome biogenesis (e.g., RimP, RplQ, RplS, RpsF, and YhbY), antibiotic responses (e.g., AmiA, EfeO, MacB, PhoU, and YcbB), post-transcriptional regulation of gene expression (e.g., Hfq, ProQ, RppH, RplC, and RpsQ), and iron-sulfur cluster assembly (e.g., ErpA, IscA, IscS, IscU, and SufA) (Figure 6B; Table 6).

## 3. Discussion

The ability of enterobacteria to colonize the digestive systems of mammals is dependent on their capacity to adapt and thrive in low-oxygen environments. Previous studies [1,2,3,4,5,6,7,8,9,10,11,12,13,14,15,16] of *E. coli* revealed that the transition from aerobic to microaerobic/anaerobic conditions requires a substantial reprogramming of gene expression, which greatly affects the bacterial lifestyle and major cellular functions such as metabolism, transport, and energy production. Nevertheless, many details of the underlying regulatory networks, including post-transcriptional mechanisms that coordinate *E. coli* adaptation and survival under oxygen-limited conditions, remain poorly defined and merit further analysis. 

In this study, we employed a combination of transcriptomic and proteomic approaches to analyze differences in RNA and protein abundance for *E. coli* grown in minimal medium under microaerobic versus aerobic conditions. Significantly, the *E. coli* cells were grown in a Bench-Top Fermentor, allowing us to control key parameters (i.e., temperature, composition of the medium, oxygen level, and pH) and thereby ensuring that growth conditions were equivalent for each experimental culture. 

Our transcriptomic analysis of aerobic and microaerobic cultures uncovered numerous upregulated and downregulated genes. Annotation and functional clustering of DEGs using the web-based tools available on the STRING, GO, KEGG, UniProtKB, RegulonDB, and EcoCyc internet platforms revealed that oxygen level directly influences carbon metabolism, energy production, metal ion homeostasis, and cell envelope functions. 

Previous studies have shown that the generation of proton motive force by cytochrome *bo* oxidase facilitates ATP production by *E. coli* ATP synthase under aerobic conditions [49]. However, this process can become less efficient at low oxygen concentrations, potentially requiring the action of a second cytochrome oxidase (i.e., cytochrome *bd-1* oxidase) with a much higher affinity for molecular oxygen. Indeed, our transcriptomic and proteomic data clearly show that the *cydABX* operon, which encodes this secondary oxidase, was strongly upregulated in *E. coli* grown under microaerobic conditions (see Table 1). Moreover, the same operon hosts another upregulated gene, namely *ndh*, which encodes NADH ubiquinone oxidoreductase (a co-factor of cytochrome *bd-1* oxidase), responsible for 2a ubiquinol regeneration.

ATP production through oxidative phosphorylation is mainly efficient during aerobic growth, with the TCA cycle greatly contributing to this process by producing the NADH and FADH that feed into the respiratory cycle. This latter occurs when succinate:quinone oxidoreductase (encoded by *sdhCDA*) converts succinate to fumarate and, concurrently, reduces ubiquinone to ubiquinol. Given the diminished role of oxidative phosphorylation under microaerobic conditions, the expression of genes coding for TCA cycle enzymes is likely reduced due to their repression by the FNR and ArcA TFs. Consistently, we observed downregulation of several operons coding for enzymes involved in steps 4 (*sucAB*; 2-oxoglutarate decarboxylase), 5 (*sucCD*; succinyl-CoA synthetase), 6 (*sdhCDAB*; succinate dehydrogenase), and 7 (*fumAC*; fumarase) of the TCA cycle. Furthermore, reduced production of these enzymes that host numerous Fe-S clusters implies a reduced cellular need for iron. Indeed, we found that many genes involved in iron homeostasis (*fhuACDB*, *fepA-entD*, *fes-ybdZ-entF-fepE*, *tonB*, *feoABC*) and the production of protein complexes responsible for iron incorporation into Fe-S clusters (*sufABCDSE* and *iscRSUA*) were repressed during microaerobic growth. Their repression is likely mediated by the TF Fur, which is activated under microaerobic conditions in the presence of free Fe^2+^ ions [10]. Simultaneously, Fur-dependent upregulation of *ftnA* (ca. 19-fold) and *bfr* (ca. 2.5-fold) elevates levels of the iron-storage proteins ferritin (2.4-fold) and bacterioferritin (1.6-fold), which efficiently sequester free iron atoms. Higher abundances of both these proteins are likely attributable to the lack of RyhB-mediated translational repression, since concentrations of this sRNA are extremely low during microaerobic growth (see below for further details). 

Our transcriptomic analysis also highlighted an enhanced role for mixed-acid fermentation (MAF) during microaerobic growth, arising from ArcA- and FNR-mediated upregulation. *E. coli* cells employ MAF to convert glucose into various end-products such as formate, succinate, acetate, lactate, and ethanol. We detected upregulation of *pflB* under microaerobiosis, suggesting an increased production of formic acid and its subsequent conversion to hydrogen (H_2_) and carbon dioxide (CO_2_) by the formate hydrogenlyase complex encoded by the *hycABCDEFGHI* operon, with the latter also being upregulated under oxygen-limited conditions. Moreover, H_2_ production appears to be coupled to reduction of menaquinone and the periplasmic protons responsible for the protein motive force that drives ATP production. This reaction is carried out by hydrogenase 1, which is encoded by another operon (i.e., *hyaABCDEF*) that is strongly upregulated in oxygen-poor environments. Similarly, we observed increased expression of several genes involved in the production of other known products of the MAF pathway, namely lactate (*ldhA*), ethanol and acetate (*adhE*), and succinate (i.e., *fumB* and *frdB*) (Table 1). 

Although we anticipated observing some changes in central carbon metabolism and energy production under microaerobiosis, the differential expression of some of the other major gene clusters is somewhat puzzling. For instance, the reasons for upregulation of multiple genes involved in acid (low pH) responses (i.e., *gadAXW*, *gadBC*, *hdeAB-yhiD*, *hdeD*) and oligopeptide transport (i.e., *oppABCDF*) are unclear. Since our cell cultures were continuously grown in a fermentor, the pH of the medium and its content was consistent throughout both aerobic and microaerobic growth, indicating that enhanced expression of these operons was not attributable to any other environmental factor except oxygen limitation. Thus, the low oxygen concentration in the environment may serve as a signal for *E. coli* to adapt to acidic environments and oligopeptide availability. Both these scenarios are encountered by enterobacteria upon entering the mammalian digestive system, which is characterized (at least in some regions) by low pH and the presence of oligopeptides produced from food digestion (i.e., polypeptide digestion by proteases). This observation raises an intriguing hypothesis that low oxygen concentrations might serve as a universal signal to alert bacterial cells that they have entered a host digestive system. Apart from microaerobiosis promoting expression of respiratory and acid stress response genes, we also detected clear upregulation of operons involved in biofilm formation (i.e., *csgDEFG* and *pgaABCD*) under this condition. Biofilm production could be considered an adaptive strategy allowing *E. coli* to survive in low-oxygen environments.

The expression patterns revealed by our transcriptomic analysis were largely confirmed by our proteomic data (Table 1). An unexpected exception was the regulation of the *cusCFBA* operon. Despite a decrease in the abundance of this polycistronic mRNA, the levels of each of the proteins encoded by this operon were increased (Table 1). The CusCFBA copper/silver efflux system contributes to maintaining copper homeostasis in low-oxygen environments [50]. The individual components of the tripartite CusCBA complex exist in a disassembled form to maintain the plasticity of the periplasm and its dynamic functions [51]. Although an increase in CusCFBA protein levels under microaerobic conditions is consistent with the documented role of this complex in copper tolerance at low oxygen concentrations [50], the exact transcriptional and post-transcriptional mechanisms responsible for the observed changes in *cusCFBA* expression at the RNA and protein levels are currently unknown. 

In addition, our proteomic data revealed a considerable reduction in the abundance of many ribosomal (r-) proteins under microaerobic conditions (Table 6 and Table S3). *E. coli* r-proteins are encoded by polycistronic operons, and they are normally autoregulated at the translational level [52]. This regulatory mechanism involves the respective free r-proteins binding to their own polycistronic mRNAs and inhibiting their translation (e.g., autoregulation of the *rpsJ-rplCDWB-rpsS-rplV-rpsC-rplP-rpmC-rpsQ* operon by L4). As the structures of the L4 binding sites in the polycistronic mRNA and ribosomal RNA closely mimic each other, L4 can act as an efficient inhibitor of its own mRNA only when it is present in excess relative to ribosomes and, therefore, is available for interaction with its cognate mRNA. In other words, a decrease in the concentration of ribosomes under microaerobic conditions should release r-proteins to inhibit translation of their cognate mRNAs, thereby reducing their abundance in vivo. Moreover, an additional extraribosomal function of L4 is to change the abundance of numerous mRNAs, mainly by inhibiting RNase E-dependent mRNA decay during bacterial adaptation to adverse environments [53]. Another extraribosomal function of L4 is to post-transcriptionally regulate Tna expression in the stationary phase of growth through its direct binding to the *tna* intergenic region [54]. Imbalances in ribosomal synthesis can release ribosomal proteins to perform other extraribosomal functions [55]. Thus, under microaerobic growth conditions, the abundance of many ribosomal proteins is reduced, possibly leading some free ribosomal proteins to perform other extraribosomal functions to maintain cell fitness.

Interestingly, although the FNR TF potentially activates the transcription of multiple genes in oxygen-limited environments, our data suggest that many FNR-dependent genes remain silent, apparently due to repression by other factors acting at the transcriptional and post-transcriptional levels. For example, FNR-mediated gene activation of nitrate reductase does not occur in the absence of nitrate and may additionally be inhibited by sRNAs such as RprA [56]. Indeed, we found that levels of RprA were considerably higher during microaerobic growth, supporting its role in controlling nitrate respiration. In fact, apart from TF-mediated gene expression (such as through Fur, FNR, and ArcA, among others), sRNAs are also widely employed by *E. coli* to exert post-transcriptional control. sRNAs in *E. coli* range from ~50 nucleotides (nt) (e.g., DicF; 53 nt) to >300 nt (e.g., CsrB; 369 nt) in length. However, detection of very short sRNAs by RNA-seq can be achieved only by including additional steps (i.e., specific size selection) in standard protocols, which are performed after fragmentation of the purified RNA and prior to cDNA library construction. Indeed, the shortest sRNA we detected was RdlD (66 nt), and we did not detect DicF (53 nt).

Our assessment of sRNA abundance uncovered several that were differentially expressed under microaerobic conditions, and their expression patterns were confirmed by Northern blotting. Particularly notable was the substantial decrease in RyhB concentration under those conditions. It is conceivable that the reduced abundance of this sRNA is inversely correlated with levels of its targets. Indeed, we observed higher abundances of the *sodB, ftnA*, and *bfr* mRNAs and their translational products (i.e., superoxide dismutase B and the two iron storage proteins FtnA and Bfr, respectively). However, low RyhB abundance did not similarly increase expression of other known RyhB targets located in the *iscRSUA*, *sucCDAB*, and *sdhCDAB* operons (Table 1), which are known for their roles in assimilating iron and homeostasis of that ion in many essential metabolic enzymes. In fact, we detected diminished abundance of their respective transcripts (see downregulated clusters in Table 1), which may be attributable to transcriptional repression by other global regulators such as the TFs FNR [57] and ArcA [58]. The latter regulators are known to downregulate the *sucCDAB* and *sdhCDAB* operons under microaerobic conditions. Moreover, the decreased expression of *sdhCDAB* we report was likely due to its repression by Fur, another TF that greatly impacts gene expression during anaerobic growth [10]. 

Unlike RyhB, we identified a number of sRNAs as being more abundant under microaerobic conditions (i.e., CsrB, CsrC, GcvB, and RprA) (Figure 3). CsrB and CsrC exert their regulatory functions by binding to the translational inhibitor CsrA, thereby preventing interaction of the latter with the translation initiation regions of numerous transcripts, including *pgaABCD* (see upregulated clusters in Table 1), under microaerobic conditions. Translational activation of this operon via competitive binding of CsrB and CsrC to CsrA enhances polysaccharide biosynthesis, thereby promoting biofilm formation. Similarly, CsrB- and CsrC-competitive binding mechanisms are likely involved in the translational activation of other genes (e.g., *iraD* [59] and *glgS* [60]) that are likewise upregulated under microaerobic conditions. The *iraD* gene encodes an anti-adapter protein that inhibits RssB-mediated degradation of the sigma stress factor RpoS, whereas GlgS is known as an inhibitor of cell motility [61]. CsrA often acts as a translational repressor, but it can also activate gene expression [60,61]. Although previous integrated transcriptomic data [62,63] have indicated that CsrA globally controls the levels of a large number of transcripts, the specific role of this translational repressor under microaerobic growth, i.e., when sRNAs CsrB/C are much more abundant than *csrA* mRNA (>60-fold; Figure 4C), remains to be determined. Interestingly, in comparison to the relatively short half-lives of CsrB (1.4 min) and CsrC (2.2. min) in Luria–Bertani (LB) medium [64], our substitution of rich medium (LB) with minimal medium and depletion of oxygen synergistically increased the stability of these small RNAs. Since both CsrB and CsrC are regulatory RNAs and substrates of RNase E, their increased stability could be attributable, at least in part, to lower RNase E levels under microaerobic conditions [42].

Notably, levels of another sRNA, GcvB, also increased (~1.8-fold) under microaerobic conditions. Despite the well-documented role of GcvB in downregulating the *oppABCDF* operon under aerobic conditions [65], our transcriptomic and proteomic data indicate that this sRNA does not inhibit *oppABCDF* expression under microaerobic conditions. Our finding that levels of aspartate 1-decarboxylase (PanD), a GcvB target previously reported to be involved in pantothenate biosynthesis [66], were decreased under microaerobiosis support the idea that GcvB may downregulate this biosynthetic pathway in oxygen-limited environments. That same study [66] reported other elements of the GcvB targetome, including the *csgDEFG* operon. It is conceivable that inhibited translation by GcvB—as well as by another sRNA, RprA, also upregulated under microaerobic conditions—likely results in reduced *csgDEFG* transcript levels. The notion that RprA exerts an active repressional role is supported by its contribution to inhibition of *dgcM* translation [2,33], which reduces levels of DgcM, a member of the signaling cascade that controls Curli biosynthesis.

It is well documented that SsrA RNA (also known as tmRNA) is involved in ribosome-associated quality control. It releases stalling ribosomes from truncated mRNAs lacking stop codons through a tmRNA-mediated mechanism, termed *trans*-translation, which includes peptide-tagging of incompletely synthesized polypeptides for degradation [67]. SsrA processing that leads to functional SsrA-tmRNA translation activity requires cleavage of the SsrA precursor by RNase E [68]. We found that SsrA was the most abundant sRNA under both aerobic and microaerobic growth conditions (Figure 3B,C). Moreover, its abundance was 1.8 times higher under microaerobiosis (Figure 3A), suggesting that protein quality control plays an important role in conferring cellular fitness under low-oxygen conditions.

## 4. Materials and Methods

### 4.1. Bacterial Strain and Growth Conditions

To prepare subcultures from fresh overnight cultures, *E. coli* K-12 strain MG1655 was grown overnight at 37 °C for 16 h in M9 medium supplemented with 0.4% glucose and trace elements (462.56 µM H_3_BO_3_, 34.71 µM MnCl_2_, 10.80 µM FeCl_3_, 7.72 µM ZnSO_4_, 3.16 µM CuSO_4_, 2.30 µM (NH_4_)_6_Mo_7_O_24_, 1.68 µM Co(NO_3_)_2_). The 16 h fresh overnight culture was diluted into 750 mL fresh M9 medium (to OD_460_ = 0.04 to 0.05) in a 1 L fermentation vessel chamber (Winpact Parallel Fermentation System FS-05-220, Saratoga, CA, USA). For aerobic culture conditions, air was continuously pumped into the chamber at 0.4 LPM (liters per min). For microaerobic culture conditions, oxygen levels in the chamber containing fresh M9 medium were initially decreased by supplying N_2_ at 0.4 LPM until dissolved oxygen (DO) reached 0. N_2_ was then pumped for a further 30 min before diluting the overnight culture in the chamber to OD_460_ = 0.04 to 0.05, and finally the N_2_ supply was turned off. The chamber was completely sealed and the culture was allowed to grow under microaerobic conditions without any additional gas supply. Aerobic and microaerobic cultures were both grown at 200 rpm, 37 °C, and maintained at pH 7.0 by automatic titration with sterile 1 M KOH. Cultures were harvested at OD_460_ = 0.5 to 0.6 for transcriptomic or proteomic analyses. 

For RNA half-life analysis, rifampicin at a final concentration of 50 mg/mL was used to inhibit new RNA synthesis, and an aliquot of the culture was collected for half-life determinations. In brief, 42 mL of culture from multiple biological replicates for each time-point was collected into 50 mL tubes with 7 mL (1/6 volume) of ice-cold stop solution (5% phenol and 95% ethanol (*v/v*)) for RNA isolation (see details below). Bacterial pellets were harvested following centrifugation at 4000× *g*, 4 °C for 15 min, and stored at −80 °C before use. We prepared 5 and 10 biological repeats for aerobic and microaerobic conditions, respectively.

### 4.2. RNA Isolation

Total RNA was extracted as described previously [17]. In brief, bacterial pellets were resuspended in 4 mL KJ medium (50 mM glucose, 25 mM Tris-HCl pH 8.0, 10 mM EDTA pH 8.0, 100 mM NaCl), lysed by placing into boiling 4 mL buffer (0.2 M NaCl, 20 mM Tris-HCl pH 7.5, 40 mM EDTA, 0.5% SDS), and boiled in a boiling water bath for 45 sec before adding 4 mL of acidic phenol (pH = 4.5) and mixing gently by slowly inverting the tube ~20 times. Total RNA was extracted in aqueous phase by centrifugation at 4000 *g*, 4 °C for 1 h. The RNA was precipitated in 1 volume of isopropanol and 1/10 volume of 3 M sodium acetate (pH 7.8) at −20 °C. All RNA samples were maintained in isopropanol at −20 °C before use. When RNA isolation was performed on aliquots, the same volume of culture from the same batch of biological replicates was used for protein isolation, Western blot analysis, and proteomic analysis.

### 4.3. RNA-seq Analysis

Total RNA in isopropanol was pelleted by centrifugation at 15,000 rpm for 15 min, washed with 70% ethanol, and then resuspended in DEPC H_2_O. DNase I (Thermo Fisher Scientific, Waltham, MA, USA) was used to remove genomic DNA. RNA quality was assessed using a Bioanalyzer (Agilent 2100). RNA samples with a 23S:16S rRNA ratio >1.4 were considered to be of sufficiently good quality for subsequent RNA-seq analysis. RNA sequencing was performed by the Genomics Core of the Institute of Molecular Biology (IMB, Academia Sinica, Taiwan) using a MiSeq or NextSeq 500 system (Illumina, San Diego, CA, USA). Removal of rRNAs was achieved using a Ribo-Zero™ Magnetic Kit (Gram-negative bacteria) in accordance with the manufacturer’s instructions (Illumina). RNA fragmentation and cDNA library construction were performed using an Illumina Stranded Total RNA kit. A BluePippin 2% gel system was used for size selection. cDNAs of 150–600 base pairs were cut from the gels and purified using a Qiagene minElute PCR kit (Qiagene, Germantown, MD, USA). Samples O-1 to O-4 and N-1 to N-4 were sequenced for 150 cycles on the MiSeq system using an Illumina MiSeq Reagent kit. An Illumina NextSeq 500 Mid Output kit was used to sequence samples O-5 and N-5 to N-10 for 150 cycles on the NextSeq 500 System. The raw RNA-seq data were processed by the Bioinformatics Core of IMB. In brief, the output fastq files were processed and analyzed using CLC Genomics Workbench 10.0.1 (CLC Bio, Cambridge, MA, USA; now Qiagen Digital Insights, Redwood City, CA, USA), according to its built-in default parameter settings, including sequence read trimming and filtering, quality control, and read mapping to a reference genome of *E. coli* (NC_000913), gene identification, reads per gene quantification, and PCA analysis. The corresponding data have been deposited to NCBI GEO (accession #GSE189154).

### 4.4. Northern Blotting

For Northern blot analysis, RNA was separated on a 7 M urea gel with 6% or 8% polyacrylamide (acrylamide/bis-acrylamide 19:1) in 0.5 × TBE and electrophoresed at 120 V until the xylene cyanide dye had reached 3/4 of the length of the gel. The RNA was transferred onto Zeta-Probe^®^Blotting membranes (Bio-Rad, Hercules, CA, USA) at 400 mA (100 min at 4 °C) in 0.5 × TBE buffer and crosslinked to the membrane using a Stratalinker 2400 UV Crosslinker (Stratagene). The membrane was pre-blotted with ULTRAhyb™ Ultrasensitive Hybridization Buffer or ULTRAhyb™-Oligo hybridization buffer (Invitrogen) for 2 h at 65 or 42 °C, respectively. An in vitro T7 transcribed [α-^32^P] UTP internally labeled RNA probe or an antisense 5′-end [γ-^32^P]-labeled DNA oligo probe was used to detect target RNA. Probe sequences are listed in Table S3. A MEGAscript™ T7 Transcription Kit (Invitrogen) or T4 polynucleotide kinase (NEB) was adapted to generate isotope-labeled probes with either [α-^32^P] UTP or [γ-^32^P] ATP. Radioactive probes were purified through a MicroSpin G-25 column (GE Healthcare) before being added into the hybridization buffer for RNA detection at 65 or 42 °C, respectively, for 6 h. Wash solution (2 × or 0.5 × SSC, 0.1% SDS) was used to remove nonspecific signals. Northern blot signals were captured by means of super-resolution BAS Storage Phosphor Screening (GE Healthcare) and detected using a GE Amersham Typhoon system.

### 4.5. Western Blot

For Western blot analysis, bacterial pellets from aerobic or microaerobic cultures were harvested at OD_460_ = 0.5 to 0.6, as described above, resuspended in 1 × sample buffer (50 mM Tris-HCl pH 6.8, 2% SDS, 12.5% glycerol, 0.001% Bromophenol blue, 2.5% 2-mercaptoethanol), and heated at 95 °C for 7 min. Samples were cooled on ice and separated on a 20% Bis-Tris SDS polyacrylamide gel at 100 V in low-molecular-weight running buffer (50 mM MES, 50 mM Tris, 1 mM EDTA, 0.1% SDS, 5 mM sodium bisulfite). After separation, proteins were transferred onto 0.45 μm PVDF immobilon-*p* membranes (Millipore, Burlington, MA, USA) using a Mini Trans-Blot Cell system (Bio-Rad) at 100 V with a constant current of 400 mA for 90 min at 4 °C in 1 × transfer buffer (25 mM Tris pH 8.3, 192 mM glycine, 20% methanol, and 0.1% SDS). Membranes were blocked with 6% milk for 1 h at room temperature and then washed with 1 × TBST buffer (20 mM Tris, 150 mM NaCl, 0.05% Tween-20 pH 7.5) for 5 min at room temperature. The membranes were hybridized with individual primary antibody for 1 h at room temperature. α-Hfq antibody (produced in our laboratory, rabbit anti-His-tag Hfq purified protein) was diluted at 1:10,000, whereas α-GAPDH monoclonal antibody (SignalChem, Richmond, VA, USA) was diluted at 1:1000. Membranes were washed once with 1 × TBST buffer for 5 min, before they were incubated with secondary antibody. Secondary mouse- or rabbit-HRP antibody (GE Healthcare, Chicago, IL, USA) was diluted at 1:10,000 and incubated for 1 h at room temperature. Finally, membranes were washed three times with 1 × TBST buffer for 5 min at room temperature. Signals were detected by means of an ECL Western Blotting Detection kit (GE Healthcare) and captured by a BioSpectrum 815 system (UVP).

### 4.6. Protein Extraction, iTRAQ Labeling, and LC-MS/MS

Sample proteins (O-1 and O-2 for aerobiosis; N2 and N-3 for microaerobiosis) were purified using a commercialized B-PER^®^ Bacterial Protein Extraction Reagent (Thermo Scientific) according to the manufacturer’s instructions. Extracted protein digestion, iTRAQ labeling of peptides and subsequent LC-MS/MS analysis, iTARQ signal normalization, and protein quantitation were performed by the core services of the Mass Spectrometry Facility (Academia Sinica, Taipei, Taiwan), as previously described [69]. Briefly, the extracted proteins were subjected to trypsin digestion at 37 °C overnight, lyophilized, and then reconstituted in iTRAQ reaction buffer. Equal amounts of peptides from each sample were individually labeled by adding iTRAQ Reagent 113, iTRAQ Reagent 114, iTRAQ Reagent 117, or iTRAQ Reagent 118, and vortexing the resulting mixtures at room temperature for 1 h. The iTRAQ-labeled peptides were then desalted using a ZipTip concentrator (Merck, Kenilworth, NJ, USA) and mixed. The multiplexed samples were further analyzed by LC-ESI-Q-TOF mass spectrometry. The resulting MS/MS spectra were exported using Mascot Distiller with default parameters. Mascot search results that satisfied the standard criteria [69] revealed the qualified peptides. Their normalized iTRAQ signals were used to quantify the relative abundances of each peptide as well as their fold-changes.

## Figures and Tables

**Table 1:** Classification of the main gene clusters differentially expressed under microaerobic versus aerobic conditions.

Upregulated Genes								
Cluster	Operon		Functional Subcategory	Transcriptional Regulator *			Translational Regulator *	
				Activator	Inhibitor	Dual **	Activator	Inhibitor
Anaerobic respiration/fermentation	Gene name	***hyaABCDE**F*	Hydrogenase	AppY, **ArcA**, YdeO	Fis, IscR, NarL, NarP	-	-	-
	RNA log_2_ fold change	**3.89_4.15_3.18_2.33_2.66** _1.99						
	Protein ratio	X_1.39_X_X_X_X						
	Gene name	***hycABCDE**F*GHI	Energy production/transport	FhlA, IHF, ModE	NsrR	-	-	-
	RNA log_2_ fold change	**4.82_3.71_2.95_3.17_2.53**_1.94_X_X_X						
	Protein ratio	X_X_X_X_X_X_X_X						
Biofilm formation, gastrointestinal tract adaptation	Gene name	***csgDEF**G *	Curli assembly	BasR, BolA, Cra, Crp, CsgD, IHF, MlrA, OmpR, ppGpp, RcdA	-	-	-	GcvB, McaS, OmrA, OmrB, RprA, RybB, RydC, Hfq, Rne
	RNA log_2_ fold change	**3.19_4.58_4.10**_X						
	Protein ratio	X_X_X_X						
	Gene name	***pgaABC**D*	Synthesis of polysacharides	Nac, NhaR	OmpR	-	-	CsrA
	RNA log_2_ fold change	**2.42_2.36_2.68**_X						
	Protein ratio	X_X_X_X						
	Gene name	***oppABCDF***	Oligopeptide transport	Nac	**Fur**, Lrp, ModE	**ArcA**	spermidine	GcvB, Hfq
	RNA log_2_ fold change	**3.09_2.98_2.86_2.75_2.71**						
	Protein ratio	**1.60**_1.10_**1.67_1.93**_X						
Acid stress resistance	Gene name	***gadAX; gadX**W*	Acid stress regulators	AdiY, **ArcA**, GadE-RcsB, GadX, PhoB, ppGpp	Nac, CRP, Fis, **FNR**, H-NS, RcsB, RutR, TorR	GadW	GadY	-
	RNA log_2_ fold change	**6.25_2.48; 2.48** _1.64						
	Protein ratio	**1.68**_X; X_X						
	Gene name	***gadBC***	Resistance to low pH	AdiY, GadE, GadX, RcsB, ppGpp	Lrp, CRP, Fis, FliZ	GadW	-	-
	RNA log_2_ fold change	**5.35_4.69**						
	Protein ratio	**2.11_1.53**						
	Gene name	***hdeAB-yhiD***	Periplasmic acid stress chaperones	GadE, RcsB, PhoP, ppGpp, TorR	-	GadW, GadX	-	-
	RNA log_2_ fold change	**5.42_5.84_5.21**						
	Protein ratio	**1.79_2.92**_X						
	Gene name	***hdeD***	Acid resistance protein	GadE, GadX, RcsB, PhoP, ppGpp	H-NS	-	-	CyaR, RprA, Hfq
	RNA log_2_ fold change	**5.87**						
	Protein ratio	X						
Microaerobic respiration	Gene name	***cydAB***	Cytochrome biosynthesis	Nac, ArcA, Cra, HypT	H-NS	FNR	-	-
	RNA log_2_ fold change	**2.74_2.74**						
	Protein ratio	**2.19** _1.45						
	Gene name	***cydX**-ybgE*	Energy production/transport	Nac	-	-	-	-
	RNA log_2_ fold change	**2.83** _1.98						
	Protein ratio	X_X						
**Downregulated genes**								
Cluster	Operon		Functional subcategory	Transcriptional regulator			Translational regulator	
				activator	inhibitor	dual*	activator	inhibitor
Cation efflux	Gene name	***cusCFBA***	Copper/silver efflux system	CusR, HprR, PhoB	-	-	-	-
	RNA log_2_ fold change	**−4.77_−4.54_−4.32_−3.40**						
	Protein ratio	1.18_**1.72**_1.36_**1.53**						
Iron homeostasis	Gene name	***fhuACDB***	Fe^3+^ transport	-	**Fur**	-	-	-
	RNA log_2_ fold change	**−3.84_−2.87_−2.45_−2.01**						
	Protein ratio	X_−1.30_X_X						
	Gene name	***fepA**-entD*	Enterobactin transporter	CRP	**Fur**	-	-	OmrA, OmrB
	RNA log_2_ fold change	**−3.37**_X						
	Protein ratio	X_X						
	Gene name	***fes-ybdZ-entF** -fepE*	Fe acquisition/incorporation of metal ions	**FNR**, H-NS	**Fur**	-	-	-
	RNA log_2_ fold change	**−5.04_−4.96_−3.10** _ − 1.69						
	Protein ratio	X_X_X_X						
	Gene name	***tonB***	Fe acquisition/energy production/siderophore, colicin, bacteriocin transport	-	-	**Fur**	**-**	-
	RNA log_2_ fold change	**−3.16**						
	Protein ratio	X						
	Gene name	***feoABC***	Fe^2+^ transport	**FNR**, OmpR	**ArcA, Fur**	NagC	-	-
	RNA log_2_ fold change	**−3.05_−3.13_−2.68**						
	Protein ratio	X_X_X						
Sulfur-involving pathways	Gene name	***sufABC**DSE*	Fe-S transport protein in Fe-S cluster assembly	IHF, IscR, OxyR, ppGpp	**Fur**, NsrR	-	-	-
	RNA log_2_ fold change	**−2.35_−2.17_−2.15**_−1.81_−1.64_X						
	Protein ratio	**−1.78** _−1.09_−1.27 _1.08 _−1.20_−1.05						
	Gene name	***iscRSU**A*	Fe-S cluster biogenesis	IscR	-	-	-	FnrS, RyhB, Hfq
	RNA log_2_ fold change	**−2.13**_−1.67_−1.58_X						
	Protein ratio	− 1.26**_−1.76_** **− 1.58_ − 1.58**						
Aerobic respiration	Gene name	***sdh**CD**AB-sucABCD-**sdhX*	TCA cycle I	Nac, CRP, **Fur**	**FNR**	**ArcA**	-	RybB, RyhB, Spf
	RNA log_2_ fold change	X_−1.65**_−2.00_−2.01_−2.02_−2.01_−2.08_−2.15**_X						
	Protein ratio	−1.30_−1.12_−1.11_−1.01_**−1.80**_−1.33_−1.15_−1.41_X						
De novo synthesis of nucleosides	Gene name	***nrdHIEF***	Nucleotide and nucleoside conversions	IscR	**Fur**, NrdR	-	-	-
	RNA log_2_ fold change	**−5.89_−4.29_−3.43_−4.20**						
	Protein ratio	X_X_X_−1.41						

Upregulated genes/operons are in red. Bold letters indicate that their RNA log_2_ fold change or protein ratio under microaerobic versus aerobic conditions were ≧ 2 or ≥ 1.5, respectively. Downregulated genes/operons are in green. Bold letters indicate that their RNA log_2_ fold change, or protein ratio under microaerobic versus aerobic conditions were ≤ −2 or ≤ −1.5, respectively; Undetected transcripts or proteins are in black or indicated by ‘X’, respectively. *: according to the RegulonDB & EcoCyc databases. **: dual regulator: a regulatory factor that, depending on the presence of other factors, can have either positive or negatively effect on gene expression. -: no reported record in the RegulonDB & EcoCyc databases.

**Table 2:** Known prophage and phage related gene operons up- and down-regulation under microaerobic versus aerobic conditions.

Prophage	Operon/Gene name	RNA (Up/Down)	Protein (Increased/Decreased)	Transcriptional Regulator *			Translational Regulator *		
				Activator	Inhibitor	Dual	Activator	Inhibitor	Attenuator
DPL12	***appY***	**1.84**	X	ArcA	DpiA, H-NS	--	--	--	--
	***ompT***	0.37	**2.07**	Lrp, PhoP	Nac	--	--	OmrA, OmrB, Hfq	--
	***rzoD***	**3.02**	X	--	--	--	--	--	--
	***ybcL** M*	**1.52** _1.36	X_X	--	--	--	--	--	--
	***ybcV***	**1.67**	X	--	Nac	--	--	--	--
	***ybcW***	**1.79**	X	--	--	--	--	--	--
	***ylcH***	**2.03**	X	--	--	--	--	--	--
e14	***ymf**TLMN**R**_beeE_jayE_ymfQ_**ycfK**-tfaP*	0.71_0.91_0.78_X_**2.34**_1.27_X_0.62**_1.86**_1.36	X_X_X_X_X_X_X_X_X_X	--	--	--	--	--	--
Qin	***cspI***	**2.63**	X	BasR	Fis	--	--	--	--
	***pinQ***	**2.36**	X	--	Nac	--	--	--	--
	***ydfV***	**4.79**	X	--	--	--	--	--	--
Rac	***kilR_ydaE***	**2.32_2.04**	X_X	--	--	--	--	--	--
	***sieB***	**2.24**	X	--	--	--	--	--	--
	***ydaG** F*	**1.53**_X	X_X	--	--	--	--	--	--
	***ynaE***	**2.23**	X	GlaR	--	--	--	--	--
phage related	***fhuA(CDB)***	**−3.84_(−2.87_−2.45_−2.01)**	X_(−1.30_X_X)	--	Fur	--	--	--	--

RNA (up/down) showing the fold change log_2_ value. Protein (increased/decreased) showing the abundance ratio value. Undetected transcripts or proteins are in black or indicated by “X”, respectively. Non-prophage related gene marking within Table 2. 2.4. Genes Encoding Transcription Factors.

**Table 3:** Higher differential expression of mRNA coding for *E. coli* transcription factors (TF).

TF	RNA (Up/Down)
CusR	−1.51
Dps	1.13
EvgA	1.3
FecI	−3.15
Fis	−1.11
GadW	1.64
GadX	2.48
IscR	−2.13
LeuO	2.21
PdhR	1.16
PutA	1.14
SoxS	−1.12

Log_2_ fold change values for up- and down-regulation mRNA are in red and green, respectively.

**Table 4:** Differential expression of genes that belong to the *E. coli* IscR regulon.

IscR Regulated Operon/Genes	RNA (Up/Down)	Protein (Increased/Decreased)
***hyaABCDE** F*	**3.89_4.15_3.18_2.33_ 2.65** _ 1.99	100 *_1.40_X_X_X_X
***sufABC**DSE*	**−2.35_−2.17_−2.15**_−1.81_−1.64_X	**− 1.78** _ − 1.09_ − 1.27 _1.08 _ − 1.20_ − 1.05
***ydiU***	**−2.01**	X
***nrdHIEF***	**−5.89_−4.29_−3.43_−4.20**	X_X_X_−1.41
*torT*	1.67	X
***iscR**SUA*	**−2.13**_−1.67_−1.58_X	− 1.26 _ **− 1.76_ − 1.58_ − 1.52**

Log_2_ fold change values for up- and down-regulated mRNA and abundance ratio value of increased and decreased protein (in red and green, respectively) are indicated. Undetected molecules are marked by “X”. *: No detected value under aerobic condition leading to a huge protein abundance ratio that is marked as “100”.

**Table 5:** Differential expression of genes that belong to the *E. coli* Fur regulon.

Fur- Fe^2+^ Regulated Operon	RNA (Up/Down)	Protein (Increased/Decreased)
***ftnA***	**4.27**	**2.4**
*fumB*	1.7	X
*ompF*	0.64	−1.62
***sdh** CD**AB-sucABC**D*	−1.51_−1.65_−2.00_−2.01_−2.02_−2.01_−2.08_−2.15	−1.30_−1.12_−1.11_−1.01_−1.80_−1.33_−1.15_−1.41
***tonB***	**−3.16**	X
***entS***	**−4.65**	X
*gdhA*	1.1	1.92
***glnK-amtB***	**3.70_3.42**	**4.42_3.94**
*aspC*	0.01	1.73
***cirA***	**−2.5**	1.27
***entCEBAH***	**−7.21_−6.18_−5.70_−4.07_−3.90**	−1.49_X_X_X_−1.17
***exbBD***	**−4.20_−4.18**	−1.60_−1.11
***exbD***	**−4.18**	−1.11
***fecIR***	**−3.15_−2.58**	X_X
***feoABC***	**−3.05_−3.13_−2.68**	X_X_X
***fepA** -entD*	**−3.37** _−0.98	X_X
***fepB***	**−3.91**	X
***fepDGC***	**−3.96_−3.37_−4.44**	X_X_X
***fes-ybdZ-entF-** fepE*	**−5.04_−4.96_−3.10** _−1.69	X_X_X_X
***fhuACDB***	**−3.84_−2.87_−2.45_−2.01**	X_−1.30_X−X
***fhuE***	**−3.12**	X
***fhuF***	**−6.12**	X
*fumC*	−1.6	−1.59
*hmp*	0.6	1.65
***nac***	3.7	X
***ndh***	**2.21**	1.32
***nrdHIEF***	**−5.89_−4.29_−3.43_−4.20**	X_X_X_−1.41
***oppABCDF***	**3.09_2.98_2.86_2.75_2.71**	1.60_1.10_1.67_1.93_X
***ryhB***	**−3.04**	X
***sodA***	**−5.8**	**−2.2**
***sufABC** DSE*	**−2.35_−2.17_−2.15** _−1.81_−1.64_−1.57	−1.78_−1.09_−1.27_1.09_−1.20_−1.05
***yjjZ***	**−2.03**	X
***yqjH***	**−3.4**	−1.29

Log_2_ fold change values for up- and down-regulated mRNA and abundance ratio value of increased and decreased protein (in red and green, respectively) are indicated.

**Table 6:** Functional classification of increased and decreased proteins.

Increased Proteins					
Biological Function	Operon		Translational Regulator *		
			Activator	Inhibitor	Attenuator
ATP metabolic process	Gene name	***cydAB***	--	--	--
	Protein ratio	**2.19** _1.45			
	RNA log_2_ fold change	**2.74_2.74**			
	Gene name	***ldhA***	--	--	--
	Protein ratio	**1.93**			
	RNA log_2_ fold change	1.97			
	Gene name	***atp**B**E**F**HA**G**DC***	--	--	--
	Protein ratio	1.28_**1.57**_1.45**_1.67_1.50**_1.43**_1.67_1.63**			
	RNA log_2_ fold change	0.29_0.14_0.20_0.18_0.20_0.17_0.17_0.45			
	Gene name	*epd -**pgk-fbaA***	--	--	--
	Protein ratio	−1.13 **_1.54_1.50**			
	RNA log_2_ fold change	−0.10 _0.82_0.70			
	Gene name	***gpmI**-envC-yibQ*	--	--	--
	Protein ratio	**1.68**_X_X			
	RNA log_2_ fold change	0.62 _−0.22_−0.33			
	Gene name	***eno***	--	--	--
	Protein ratio	**1.80 **			
	RNA log_2_ fold change	1.35			
	Gene name	***tpiA***	--	--	--
	Protein ratio	**1.73**			
	RNA log_2_ fold change	0.51			
	Gene name	***pfkA***	--	--	--
	Protein ratio	**1.53**			
	RNA log_2_ fold change	0.35			
	Gene name	***pfkB***	--	--	--
	Protein ratio	**1.50 **			
	RNA log_2_ fold change	0.27			
	Gene name	***glk***	--	--	--
	Protein ratio	**1.91**			
	RNA log_2_ fold change	0.75			
Pyruvate metabolic process	Gene name	***ispDF***	--	--	--
	Protein ratio	**1.96** _1.09			
	RNA log_2_ fold change	− 0.39_ − 0.27			
	Gene name	*epd -**pgk-fbaA***	--	--	--
	Protein ratio	−1.13 **_1.54_1.50**			
	RNA log_2_ fold change	−0.10 _0.82_0.70			
	Gene name	***gpmI**-envC-yibQ*	--	--	--
	Protein ratio	**1.68**_X_X			
	RNA log_2_ fold change	0.62 _−0.22_−0.33			
	Gene name	***eno***	--	--	--
	Protein ratio	**1.80 **			
	RNA log_2_ fold change	1.35			
	Gene name	***tpiA***	--	--	--
	Protein ratio	**1.73**			
	RNA log_2_ fold change	0.51			
	Gene name	***pfkA***	--	--	--
	Protein ratio	**1.53**			
	RNA log_2_ fold change	0.35			
	Gene name	***pfkB***	--	--	--
	Protein ratio	**1.50 **			
	RNA log_2_ fold change	0.27			
	Gene name	***glk***	--	--	--
	Protein ratio	**1.91**			
	RNA log_2_ fold change	0.75			
Glycolytic process	Gene name	*epd -**pgk-fbaA***	--	--	--
	Protein ratio	−1.13 **_1.54_1.50**			
	RNA log_2_ fold change	−0.10 _0.82_0.70			
	Gene name	***gpmI**-envC-yibQ*	--	--	--
	Protein ratio	**1.68**_X_X			
	RNA log_2_ fold change	0.62 _−0.22_−0.33			
	Gene name	***eno***	--	--	--
	Protein ratio	**1.80 **			
	RNA log_2_ fold change	1.35			
	Gene name	***tpiA***	--	--	--
	Protein ratio	**1.73**			
	RNA log_2_ fold change	0.51			
	Gene name	***pfkA***	--	--	--
	Protein ratio	**1.53**			
	RNA log_2_ fold change	0.35			
	Gene name	***pfkB***	--	--	--
	Protein ratio	**1.50 **			
	RNA log_2_ fold change	0.27			
	Gene name	***glk***	--	--	--
	Protein ratio	**1.91**			
	RNA log_2_ fold change	0.75			
Glucose metabolic process	Gene name	***sdaA***	--	--	--
	Protein ratio	**2.07**			
	RNA log_2_ fold change	0.78			
	Gene name	*focA**-pflB***	--	--	--
	Protein ratio	X**_2.03**			
	RNA log_2_ fold change	1.01_1.47			
	Gene name	***pckA***	--	--	--
	Protein ratio	**1.60 **			
	RNA log_2_ fold change	0.69			
	Gene name	*epd -**pgk-fbaA***	--	--	--
	Protein ratio	−1.13 **_1.54_1.50**			
	RNA log_2_ fold change	−0.10 _0.82_0.70			
	Gene name	***gpmI**-envC-yibQ*	--	--	--
	Protein ratio	**1.68**_X_X			
	RNA log_2_ fold change	0.62 _−0.22_−0.33			
	Gene name	***tpiA***	--	--	--
	Protein ratio	**1.73**			
	RNA log_2_ fold change	0.51			
	Gene name	***pfkA***	--	--	--
	Protein ratio	**1.53**			
	RNA log_2_ fold change	0.35			
Purine-containing compound metabolic process	Gene name	*allDC-**ylbA***	--	--	--
	Protein ratio	X-X**−1.51**			
	RNA log_2_ fold change	0.25_0.47_0.56			
	Gene name	*atpBEFHAGDC*	--	--	--
	Protein ratio	1.28_**1.57**_1.45**_1.67_1.50**_1.43**_1.67_1.63**			
	RNA log_2_ fold change	0.29_0.14_0.20_0.18_0.20_0.17_0.17_0.45			
	Gene name	***ackA** -pta*	--	SdhX	--
	Protein ratio	**1.99** _−1.13			
	RNA log_2_ fold change	1.19–0.97			
	Gene name	*epd -**pgk-fbaA***	--	--	--
	Protein ratio	−1.13 **_1.54_1.50**			
	RNA log_2_ fold change	−0.10 _0.82_0.70			
	Gene name	***gpmI**-envC-yibQ*	--	--	--
	Protein ratio	**1.68**_X_X			
	RNA log_2_ fold change	0.62 _−0.22_−0.33			
	Gene name	***eno***	--	--	--
	Protein ratio	**1.80 **			
	RNA log_2_ fold change	1.35			
	Gene name	***tpiA***	--	--	--
	Protein ratio	**1.73**			
	RNA log_2_ fold change	0.51			
	Gene name	***pfkA***	--	--	--
	Protein ratio	**1.53**			
	RNA log_2_ fold change	0.35			
	Gene name	***pfkB***	--	--	--
	Protein ratio	**1.50 **			
	RNA log_2_ fold change	0.27			
	Gene name	***glk***	--	--	--
	Protein ratio	**1.91**			
	RNA log_2_ fold change	0.75			
Ion homeostasis	Gene name	***gadA**X*	GadY (onto GadX)	--	--
	Protein ratio	**1.68**_X			
	RNA log_2_ fold change	**6.25_2.48**			
	Gene name	***cus**C**FBA***	--	--	--
	Protein ratio	1.18**_1.72**_1.36**_1.53**			
	RNA log_2_ fold change	**−4.77_−4.54_−4.32_−3.40**			
	Gene name	***copA***	--	--	--
	Protein ratio	**1.67**			
	RNA log_2_ fold change	−1.11			
	Gene name	***chaA***	--	--	--
	Protein ratio	**1.77**			
	RNA log_2_ fold change	0.55			
	Gene name	***gadBC***	--	--	--
	Protein ratio	**2.12_1.53**			
	RNA log_2_ fold change	**5.35_4.69**			
	Gene name	*bfd-**bfr***	--	RyhB	--
	Protein ratio	X_**1.59**			
	RNA log_2_ fold change	**−3.99** _1.32			
	Gene name	***adiA***	--	--	--
	Protein ratio	**1.78**			
	RNA log_2_ fold change	0.31			
	Gene name	***ftnA***	--	--	--
	Protein ratio	**2.40 **			
	RNA log_2_ fold change	**4.27 **			
**Decreased proteins**					
Biological function	Operon		Translational regulator *		
			activator	inhibitor	attenuator
Ribosome assembly	Gene name	***rpsB** -tsf*	--	RpsB	--
	Protein ratio	**−1.99** _ −1.03			
	RNA log_2_ fold change	−0.28_−0.21			
	Gene name	***rpsMK**D-rpoA-**rplQ***	--	RpsD	--
	Protein ratio	**−1.67_−1.54_−1.55** _−1.17**_−1.81**			
	RNA log_2_ fold change	−0.51_−0.63_−0.61_−0.61_−0.70			
	Gene name	*metY-**yhbC**-nusA-infB-rbfA-truB-**rpsO**-pnp*	--	--	--
	Protein ratio	X**_−1.70**_−1.31_−1.38_−1.22_−1.21**_−1.77**_−1.12			
	RNA log_2_ fold change	0.29 _−0.50_−0.39_−0.39_−0.20_−0.56_−0.50_−0.45_−0.38			
	Gene name	***rpsJ-rplCDWB-rpsS-rplV-rpsC**-rplP-**rpmC-rpsQ***	--	--	RplD
	Protein ratio	**−1.81_−1.88_−1.98_−1.97_−1.97_−1.94_−1.67_−1.84** _−1.47**_−1.57_−1.76**			
	RNA log_2_ fold change	−0.67_−0.84_−0.89_−0.80_−0.92_−0.98_−0.86_−0.71_−0.67_−0.66_−0.78			
	Gene name	***rplNXE-rpsNH-rplFR-rpsE-rpmD-rplO** -secY-**rpmJ***	--	RpsH	--
	Protein ratio	**−1.67_−1.68_−1.99_−2.08_−1.57_−1.78_−1.64_−1.63_−1.84_−1.73** _−1.13**_−1.54**			
	RNA log_2_ fold change	−0.36_−0.70_−0.62_−0.60_−0.78_−0.70_−0.69_−0.70_−0.62_−0.70_−0.60_−0.34			
	Gene name	***rpsU**-dnaG-rpoD*	--	--	--
	Protein ratio	**−1.87**_X_1.38			
	RNA log_2_ fold change	−0.70_−0.36_−0.16			
	Gene name	***rpsA** -ihfB*	--	RpsA	--
	Protein ratio	**−1.82**_−1.39			
	RNA log_2_ fold change	−0.46_−0.00			
	Gene name	***rpsT***	--	--	--
	Protein ratio	**−1.55**			
	RNA log_2_ fold change	−0.51			
	Gene name	***rpsLG**-fusA-tufA*	--	RpsG	--
	Protein ratio	**−1.77_−1.68**_−1.14_X			
	RNA log_2_ fold change	−0.42_−0.47_−0.63_−0.35			
	Gene name	***rplK** AJL-rpoBC*	--	RplA	--
	Protein ratio	**−1.55** _−1.49_−1.42_−1.44_−1.13_−1.12			
	RNA log_2_ fold change	−0.50_−0.52_−0.65_−0.74_−0.43_−0.46			
	Gene name	***yhbY***	--	--	--
	Protein ratio	**−1.67**			
	RNA log_2_ fold change	−0.36			
	Gene name	*thrS-**infC**-rpmI-**rplT**-pheMST-ihfA*	--	RplT	--
	Protein ratio	−1.03**_−1.51**_−1.17**_−2.19**_X_1.44_1.38_−1.36			
	RNA log_2_ fold change	−0.26_−0.02_−0.03_−0.14_X_−0.07_−0.11_−0.36			
	Gene name	***rpmBG**-mutM*	--	--	--
	Protein ratio	**−1.80_−1.80**_X			
	RNA log_2_ fold change	−0.87_−1.12_−0.93			
	Gene name	***rplY***	--	RplY	--
	Protein ratio	**−2.34**			
	RNA log_2_ fold change	−0.43			
	Gene name	***rlmE** -ftsH*	--	--	--
	Protein ratio	**−4.10** _−1.09			
	RNA log_2_ fold change	−0.17_−0.25			
	Gene name	*yceD-**rpmF**;** rpmF**-plsX-fabHDG*	--	--	--
	Protein ratio	−1.81**_−1.62**; **−1.62** _1.03 _−1.17 _1.18 _−1.17			
	RNA log_2_ fold change	−0.64_−0.51; −0.51_−0.06_−0.16 _0.00 _−0.34			
	Gene name	***rplU-rpmA***	--	--	--
	Protein ratio	**−1.89_−1.61**			
	RNA log_2_ fold change	−0.65_−0.61			
Negative regulation of translation	Gene name	***hfq**-hflXKC*	--	--	--
	Protein ratio	**−1.67**_X**_1.71**_1.21			
	RNA log_2_ fold change	−0.36_−0.25_−0.28_−0.33			
	Gene name	***rplM-rpsI***	--	RplM	--
	Protein ratio	**−1.95_−1.75**			
	RNA log_2_ fold change	−0.74_−0.64			
	Gene name	***rpsLG**-fusA-tufA*	--	RpsG	--
	Protein ratio	**−1.77_−1.68**_−1.14_X			
	RNA log_2_ fold change	−0.42_−0.47_−0.63_−0.35			
	Gene name	***rpsMKD**-rpoA **-rplQ***	--	RpsD	--
	Protein ratio	**−1.67_−1.54_−1.55** _−1.17 **_1.67**			
	RNA log_2_ fold change	−0.51_−0.63_−0.61_−0.61_−0.70			
	Gene name	***rpsJ-rplCDWB-rpsS-rplV-rpsC**-rplP-**rpmC-rpsQ***	--	--	RplD
	Protein ratio	**−1.81_−1.88_−1.98_−1.97_−1.97_−1.94_−1.67_−1.84** _−1.47**_−1.57_−1.76**			
	RNA log_2_ fold change	−0.67_−0.84_−0.89_−0.80_−0.92_−0.98_−0.86_−0.71_−0.67_−0.66_−0.78			
	Gene name	***rpsA** -ihfB*	--	RpsA	--
	Protein ratio	**−1.82** _−1.39			
	RNA log_2_ fold change	−0.46_−0.00			
	Gene name	*thrS-**infC**-rpmI-**rplT**-pheMST-ihfA*	--	RplT	--
	Protein ratio	−1.03**_−1.51**_−1.17**_−2.19**_X_1.44_1.38_−1.36			
	RNA log_2_ fold change	−0.26_−0.02_−0.03_−0.14_X_−0.07_−0.11_−0.36			
	Gene name	***rplY***	--	RplY	--
	Protein ratio	**−2.34**			
	RNA log_2_ fold change	−0.43			
Response to antibiotic	Gene name	*pstSCAB**-phoU***	--	--	--
	Protein ratio	−1.41_X_X_X**_−1.85**			
	RNA log_2_ fold change	−1.02_−1.01_−0.78_−0.93			
	Gene name	***mac** A**B***	--	--	--
	Protein ratio	−1.10**_−1.82**			
	RNA log_2_ fold change	0.10 _−0.05			
	Gene name	***amiA** -hemF*	--	--	--
	Protein ratio	**−1.68** _−1.13			
	RNA log_2_ fold change	−1.66_−1.41			
	Gene name	***rpmBG**-mutM*	--	--	--
	Protein ratio	**−1.80_−1.80**_X			
	RNA log_2_ fold change	−0.87_−1.12_−0.93			
	Gene name	***efeO**B*	--	--	--
	Protein ratio	**−2.97**_X			
	RNA log_2_ fold change	**−6.19_−4.74**			
	Gene name	***ldtD/ycbB***	--	--	--
	Protein ratio	**−7.34**			
	RNA log_2_ fold change	−0.11			
	Gene name	***rpsMKD**-rpoA -rplQ*	--	RpsD	--
	Protein ratio	**−1.67_−1.54_−1.55** _−1.17 **_1.67**			
	RNA log_2_ fold change	−0.51_−0.63_−0.61_−0.61_−0.70			
	Gene name	***rpsJ-rplCDWB-rpsS-rplV-rpsC**-rplP-**rpmC-rpsQ***	--	--	RplD
	Protein ratio	**−1.81_−1.88_−1.98_−1.97_−1.97_−1.94_−1.67_−1.84** _−1.47**_−1.57_−1.76**			
	RNA log_2_ fold change	−0.67_−0.84_−0.89_−0.80_−0.92_−0.98_−0.86_−0.71_−0.67_−0.66_−0.78			
	Gene name	* **rplNXE-rpsNH-rplFR-rpsE-rpmD-rplO** -secY-**rpmJ***	--	RpsH	--
	Protein ratio	**−1.67_−1.68_−1.99_−2.08_−1.57_−1.78_−1.64_−1.63_−1.84_−1.73** _−1.13**_−1.54**			
	RNA log_2_ fold change	−0.36_−0.70_−0.62_−0.60_−0.78_−0.70_−0.69_−0.70_−0.62_−0.70_−0.60_−0.34			
	Gene name	***rpsLG**-fusA-tufA*	--	RpsG	--
	Protein ratio	**−1.77_−1.68**_−1.14_X			
	RNA log_2_ fold change	−0.42_−0.47_−0.63_−0.35			
Posttranscriptional regulation of gene expression	Gene name	***proQ** -prc*	--	--	--
	Protein ratio	**−1.68** _−1.24			
	RNA log_2_ fold change	−0.44_−0.34			
	Gene name	***rppH** -ptsP*	--	--	--
	Protein ratio	**−1.66** _−1.27			
	RNA log_2_ fold change	−0.21_−0.11			
	Gene name	***rpsMKD**-rpoA **-rplQ***	--	RpsD	--
	Protein ratio	**−1.67_−1.54_−1.55** _−1.17 _**1.67**			
	RNA log_2_ fold change	−0.51_−0.63_−0.61_−0.61_−0.70			
	Gene name	*metY-**yhbC**-nusA-infB-rbfA-truB-**rpsO**-pnp*	--	--	--
	Protein ratio	X**_−1.70**_−1.31_−1.38_−1.22_−1.21**_−1.77**_−1.12			
	RNA log_2_ fold change	0.29 _−0.50_−0.39_−0.39_−0.20_−0.56_−0.50_−0.45_−0.38			
	Gene name	***rpsJ-rplCDWB-rpsS-rplV-rpsC**-rplP-**rpmC-rpsQ***	--	--	RplD
	Protein ratio	**−1.81_−1.88_−1.98_−1.97_−1.97_−1.94_−1.67_−1.84** _−1.47**_−1.57_−1.76**			
	RNA log_2_ fold change	−0.67_−0.84_−0.89_−0.80_−0.92_−0.98_−0.86_−0.71_−0.67_−0.66_−0.78			
	Gene name	***rplNXE-rpsNH-rplFR-rpsE-rpmD-rplO** -secY-**rpmJ***	--	RpsH	--
	Protein ratio	**−1.67_−1.68_−1.99_−2.08_−1.57_−1.78_−1.64_−1.63_−1.84_−1.73** _−1.13**_−1.54**			
	RNA log_2_ fold change	−0.36_−0.70_−0.62_−0.60_−0.78_−0.70_−0.69_−0.70_−0.62_−0.70_−0.60_−0.34			
	Gene name	***rpsA** -ihfB*	--	RpsA	--
	Protein ratio	**−1.82** _−1.39			
	RNA log_2_ fold change	−0.46_−0.00			
	Gene name	***rpsLG**-fusA-tufA*	--	RpsG	--
	Protein ratio	**−1.77_−1.68**_−1.14_X			
	RNA log_2_ fold change	−0.42_−0.47_−0.63_−0.35			
	Gene name	*thrS-**infC**-rpmI-**rplT**-pheMST-ihfA*	--	RplT	--
	Protein ratio	−1.03**_−1.51**_−1.17**_−2.19**_X_1.44_1.38_−1.36			
	RNA log_2_ fold change	−0.26_−0.02_−0.03_−0.14_X_−0.07_−0.11_−0.36			
	Gene name	***rplY***	--	RplY	--
	Protein ratio	**−2.34**			
	RNA log_2_ fold change	−0.43			
	Gene name	***hfq**-hflX**K**C*	--	--	--
	Protein ratio	**−1.67**_X**_1.71**_1.21			
	RNA log_2_ fold change	−0.36_−0.25_−0.28_−0.33			
Iron-sulfur cluster assembly	Gene name	***isc** R**SUA***	--	Hfq, RyhB	--
	Protein ratio	−1.26_**−1.76_−1.58_−1.58**			
	RNA log_2_ fold change	**−2.13** _−1.67_−1.58_−1.44			
	Gene name	***erpA***	--	RyhB	--
	Protein ratio	**−1.99**			
	RNA log_2_ fold change	−0.82			
	Gene name	***sufA** BCDSE*	--	--	--
	Protein ratio	**−1.78** _−1.09_−1.27_−0.92_−1.20_−1.05			
	RNA log_2_ fold change	**−2.35_−2.17_−2.15** _−1.81_−1.64_−1.57			

Operon gene name showed red letter: increased; bold red letter with protein ratio ≥ 1.5; black letter: not detected. Operon gene name showed green letter: decreased; bold green letter with protein ratio ≤ −1.5; black letter: not detected. *: According to the RegulonDB & EcoCyc databases. --: No reported record in RegulonDB & EcoCyc databases.

## Data Availability

The RNA sequencing data and related information have been deposited to NCBI GEO (accession #GSE189154).

## Supplementary Materials

The following supporting information can be downloaded at: https://www.mdpi.com/article/10.3390/ijms23052570/s1.
