# Supplementary material for: Combined Transcriptomic and Proteomic Profiling of E. coli under Microaerobic versus Aerobic Conditions: The Multifaceted Roles of Noncoding Small RNAs and Oxygen-Dependent Sensing in Global Gene Expression Control
Source: Int J Mol Sci. 2022 Feb 25;23(5):2570. doi: 10.3390/ijms23052570 (PMC8910356; doi:10.3390/ijms23052570)
Supplement: Supplementary file 1 [file ijms-23-02570-s001.zip › FigS1_S2_TabS1_S3-S4-20220215-01.pdf]

A

sample r sample	N-1	N-2	N-3	N-4	N-5	N-6	N-7	N-8	N-9
N-2	0.981								
N-3	0.970	0.986							
N-4	0.961	0.976	0.993						
N-5	0.984	0.969	0.959	0.964					
N-6	0.976	0.946	0.936	0.945	0.996				
N-7	0.982	0.946	0.925	0.915	0.982	0.982			
N-8	0.975	0.970	0.966	0.977	0.996	0.990	0.963		
N-9	0.992	0.970	0.946	0.933	0.982	0.976	0.994	0.967	
N-10	0.979	0.950	0.920	0.915	0.984	0.985	0.993	0.968	0.991

O-4	O-3	O-2	O-1	sample r sample
0.989	0.982	0.974	0.981	O-5
	0.999	0.992	0.997	O-4
		0.993	0.998	O-3
			0.998	O-2

B

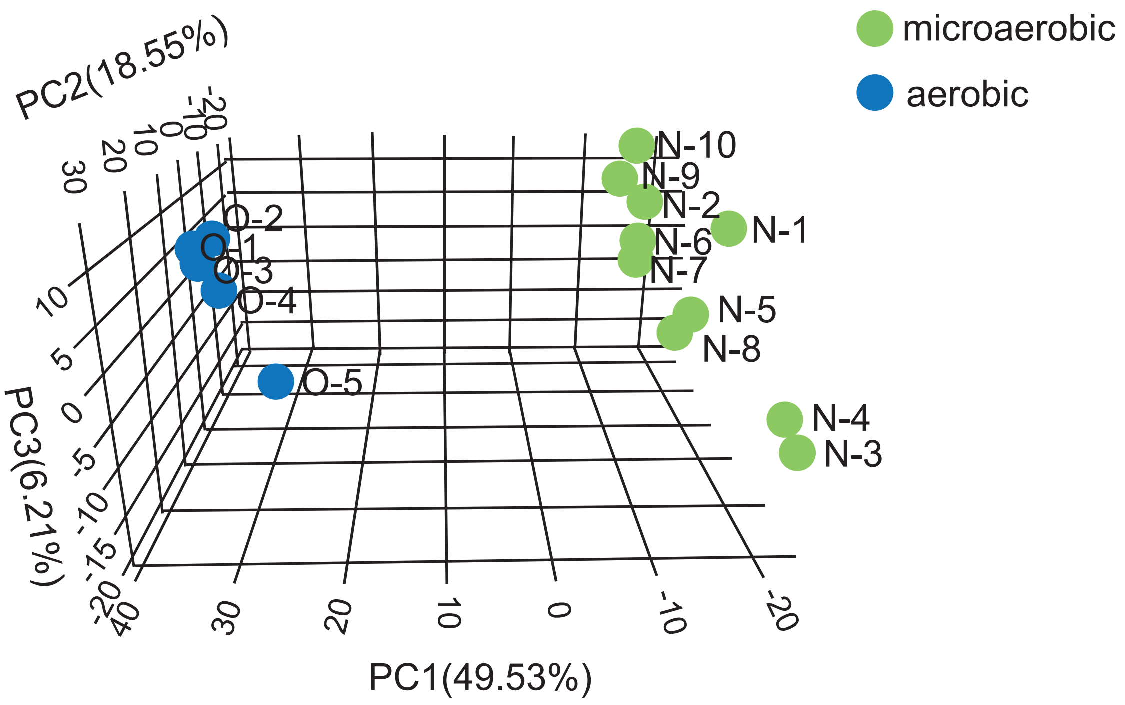

Figure S1

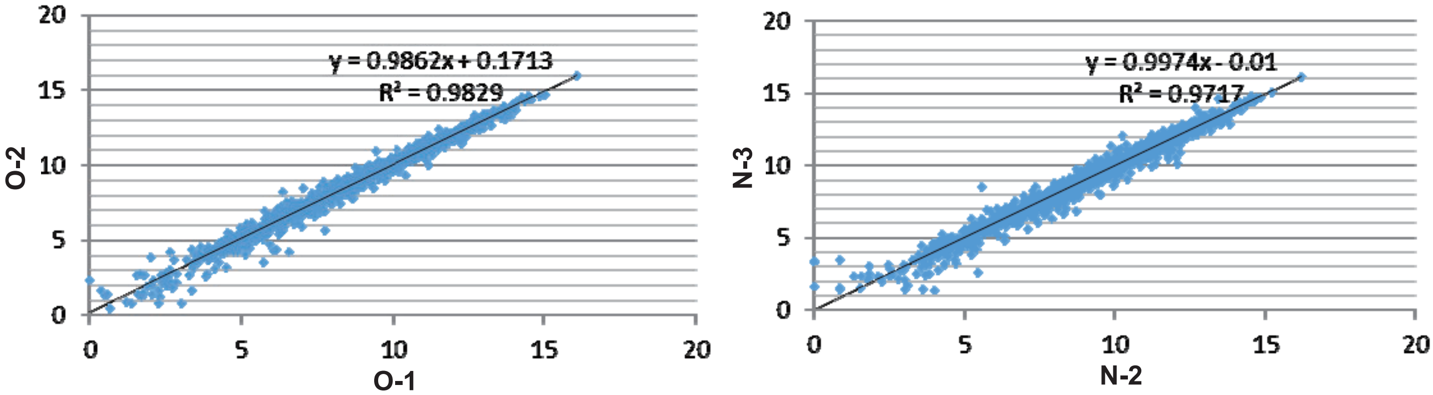

y = 0.9862x + 0.1713
R^2 = 0.9829
O-2
O-1
0
5
10
15
20
y = 0.9974x - 0.01
R^2 = 0.9717
N-3
N-2

Figure S2

Table S1. The coverage of sequence reads.

Culture condition	Average # of reads	Average counted fragments	% of total	Average unique fragments	% of total
Aerobic	10861836	10494237	95.78	10367181	94.60
Microaerobic	9304156	8448382	90.78	8271205	88.82

Table S3. Differentially expressed *E coli* K12 encoded protein identified by quantitative proteomic analysis.

protein name/ accession number*	protein abundance ratio (up / down)	protein name/ accession number	protein abundance ratio (up / down)	protein name/ accession number	protein abundance ratio (up / down)	protein name/ accession number	protein abundance ratio (up / down)	protein name/ accession number	protein abundance ratio (up / down)	protein name/ accession number	protein abundance ratio (up / down)	protein name/ accession number	protein abundance ratio (up / down)
MnmE/ C4ZYY4	5.14	YtfQ/ P39325	1.84	ArtQ/ P0AE34	1.65	YqcA/ P65367	1.54	Ffh/ P0AGD7	-4.05	RplS/ B6I628	-1.79	OmpF/ P02931	-1.62
XylA/ P00861	4.34	YciE/ P21363	1.81	Hmp/ P24232	1.65	GldA/ P0A9S5	1.54	CdaR/ P37047	-3.17	FabZ/ B5Z0F9	-1.79	MbeA/ P13658	-1.62
YebE/ P33218	3.78	Eno/ B1XDI9	1.80	FliJ/ P52613	1.65	YcfD/ P27431	1.54	CheY/ P0AE67	-3.00	SufA/ P77667	-1.78	Rim/ B1XBT0	-1.60
DadX/ P29012	3.08	YnfD/ P76172	1.80	MenC/ P29208	1.65	YbhL/ P0AAC4	1.53	EfeO/ P0AB24	-2.97	GuaA/ P04079	-1.78	Gss/ P0AES0	-1.59
YjiY/ P39396	2.88	CpxR/ P0AE88	1.80	SodB/ P0AGD3	1.64	PfkA/ B1XB82	1.53	YadS/ P0AFP0	-2.75	RplF/ P0AG55	-1.78	FumC/ P05042	-1.59
Spy/ P77754	2.73	MdtE/ P37636	1.80	YbbP/ P77504	1.63	CusA/ P38054	1.53	YfaZ/ P76471	-2.34	RpsL/ A8A5E9	-1.77	RluC/ P0AA39	-1.59
HisJ/ P0AEU0	2.63	AdiA/ P28629	1.78	AtpC/ C4ZZ09	1.63	GadC/ P63235	1.53	YiaD/ P37665	-2.27	Upp/ B7UGN6	-1.77	IscA/ C4ZXA3	-1.58
TusC/ P45531	2.62	GorA/ P28304	1.78	YdbK/ P52647	1.63	YnjB/ P76223	1.53	Soda/ P00448	-2.20	RpsO/ B1XGX7	-1.77	YeeD/ P33014	-1.58
FtnA/ P0A999	2.40	Tam/ P76145	1.78	YfcH/ P77775	1.62	AlsB/ P39265	1.53	MurE/ P22188	-2.15	YecF/ P0AD08	-1.77	IscU/ P0ACD4	-1.58
HisB/ P06987	2.31	YcaC/ P21367	1.77	PncA/ P21369	1.61	ArtM/ P0AE30	1.53	OmpC/ P06996	-2.12	RpsQ/ A1AGK0	-1.76	PtsI/ P08839	-1.57
HtpX/ B1XH97	2.30	LrhA/ P36771	1.77	PanB/ C4ZRM7	1.61	AmpE/ P0AE14	1.52	YhhZ/ P46855	-2.11	IscS/ A7ZPX4	-1.76	ProS/ B1XD64	-1.57
OsmC/ P0C0L2	2.27	LsrF/ B1XEA5	1.77	OppA/ P23843	1.60	AllE/ P75713	1.51	DhaM/ P37349	-2.02	ThrA/ P00561	-1.76	Rna/ P21338	-1.55
LeuA/ C4ZPZ7	2.24	ChaA/ P31801	1.77	YciI/ P0AB55	1.60	Acre/ P24180	1.51	MetA/ P07623	-1.99	MepA/ C4ZVM1	-1.75	CspE/ P0A972	-1.55
CysS/ C4ZUX4	2.23	HisD/ P06988	1.76	Bfr/ P0ABD3	1.59	UspC/ P46888	1.51	RplE/ P62399	-1.99	YciG/ P21361	-1.75	RpsT/ B1XBE8	-1.55
PnuC/ P0AFK2	2.19	ArtI/ P30859	1.76	QorB/ P39315	1.59	ApaH/ B1XC50	1.51	RplW/ P0ADZ0	-1.97	RpsI/ P0A7X3	-1.75	NuoF/ P31979	-1.54
CydA/ P0ABJ9	2.19	RfbC/ P37745	1.75	YdhF/ P76187	1.59	EutB/ P0AEJ6	1.50	NarP/ P31802	-1.95	Crp/ P0ACK0	-1.74	FadI/ C4ZVN3	-1.54
YcaK/ P43340	2.10	HisC/ P06986	1.74	Slp/ P37194	1.59	YedQ/ P76330	1.50	RplM/ B1XHK4	-1.95	YcbB/ P22525	-1.74	YbgC/ P0A8Z3	-1.54
OmpT/ P09169	2.07	AspC/ P00509	1.73	SstT/ P0AGE4	1.58	FbaA/ P0AB71	1.50	BcsC/ P37650	-1.92	RplO/ B1X6F3	-1.73	RpmJ/ P0A7Q6	-1.54
SdaA/ P16095	2.07	CusF/ P77214	1.72	ChrR/ P0AGE6	1.57	PfkB/ P06999	1.50	SecA/ C4ZRJ3	-1.92	RluB/ P37765	-1.73	FrvR/ P32152	-1.54
PflB/ P09373	2.03	ArsC/ P0AB96	1.72	YhhA/ P0ADX7	1.57	Gpr/ Q46851	1.50	TrmB/ P0A8I5	-1.91	YncE/ P76116	-1.73	YdiY/ P76206	-1.53
YqjG/ P42620	2.00	AdhE/ P0A9Q7	1.70	ZraP/ P0AAA9	1.56			LivK/ P04816	-1.86	ProQ/ P45577	-1.68	HemL/ B1XD24	-1.52
MoaD/ P30748	1.98	YjcH/ P0AF54	1.69	NarZ/ P19319	1.56			RspA/ P38104	-1.86	RpsF/ P02358	-1.68	LplA/ C4ZT68	-1.52
AckA/ P0A6A3	1.97	YdiH/ P64476	1.68	FrsA/ C4ZT96	1.56			Rho/ P0AG30	-1.86	FkpA/ P45523	-1.68	ThiI/ P77718	-1.52
IspD/ B1XCS3	1.96	GpmI/ P37689	1.68	YsgA/ P56262	1.56			PhoU/ P0A9K7	-1.85	AmiA/ P36548	-1.68	BWG/ C4ZU91	-1.52
GloA/ P0AC81	1.94	NarV/ P0AF32	1.68	YihX/ P0A8Y3	1.55			NuoI/ P0AFD6	-1.84	RpsG/ P02359	-1.68	GltI/ P37902	-1.51
LdhA/ P52643	1.93	MetE/ B1XAJ3	1.68	YhfA/ P0ADX1	1.55			RpmD/ C4ZUF7	-1.84	TyrS/ C4ZYA0	-1.67	YedF/ P0AA31	-1.51
OppD/ P76027	1.93	CopA/ Q59385	1.67	GatB/ P37188	1.55			RpsA/ P0AG67	-1.82	Lcd/ P08200	-1.67	YdjF/ P77721	-1.50
ArgC/ P11446	1.93	ThiD/ P76422	1.66	HisF/ P60664	1.55			RpsJ/ C4ZUH6	-1.81	YhbY/ P0AGK4	-1.67	MetF/ P0AEZ1	-1.50
GdhA/ P00370	1.92	YqjI/ P64588	1.66	Pgk/ P0A799	1.54			RplQ/ C4ZUE9	-1.81	NagE/ P09323	-1.66	Acs/ P27550	-1.50
YdbH/ P52645	1.89	YqiC/Q4 6868	1.66	LdcC/ P52095	1.54			YebO/ P64499	-1.81	YpeA/ P76539	-1.65	Sad/ P76149	-1.50
GatY/ P0C8J6	1.85	YgfZ/ B1XEI5	1.65	ArgT/ P09551	1.54			YhdV/ P64622	-1.79	GhrB/ C4ZXE2	-1.63		

*: UniPort accession number

Table S4. The list of probes used in this study.

DNA Probes	Sequence (5' to 3')
ChiX	ATA TCG CTA TTG GCC CGT CAA AGA GGA ATT
CsrB	CTG ATG TTC ACT TCG TTG TCT GAC TCC CTG TC
CsrB T7 F	GTT AAA GGA CAC CTC CAG
CsrB T7 R	TAA TAC GAC TCA CTA TAG GGT CGT TTC GCA GCA TTC CAG
CsrC-1	CCG TTT AAT TAC GTC TCC GGA CGT TTG TC
CsrC-2	TGT CGC CTT TTT TCC
GadY	GTC TCC AGA CTA ATA AAC CGT TAT AAC ACT CCC TGT TGG CAC GGG AAA CTT TGT GCT CTC
GcvB	GGA CAG ACA GGG TAA ATG TAC AGG AAG TGA AAA AAG GTA GCT TTG CTA CCA TGG TCT GAA
GlmY	AAG TTG GAC GGC AGG CAC CTT GTT GTG CGT CAT TCG
MicF	CCG GTT GAA ATA GGG GTA AAC AGA CAT TCA GAA ATG AAT GAC GG
RprA	CAC ACA GCA ATT CGT TGT TTC ACT CAG GGG ATT TCC ATG C
RybA	CAC GCG CAT ACA CCT CTT GAA CTC ATT CAT AAG ACC TCC TG
RybB	GGG CTC CAC AAA ATG GGG ACA TCA AAG AAA AGC AGT GGC
RyeA	GCG TCG TCA TCT ATT CTT AAA GGG CAA GGC AAC
RyhB	CTG GAA GCA ATG TGA GCA ATG TCG TGC TTT CAG GTTC
RyjA	GGT CTT TGT CTT TCT CTC TAT CCC GCT GGT ACA CAG G
SraF	GCC GCT GCT TTT TAT GCA TAG ACC TCG CCT TCC GG
tRNAAsn*	TAC GGA TTA ACA GTC CGC CGT TCT ACC GAC TGA ACT ACA GA
5S rRNA*	CGC TAC GGC GTT TCA CTT CTG AGT TCG GC
6S RNA*	GGT GAA TGT GTC GTC GCA GTT TTA AGG CTT CTC GGA
M1 RNA*	GCT CTC TGT TGC ACT GGT CGT GGG TTT CC

* Northern Blotting's specific DNA probes can detect tRNAAsn, 5S rRNA, 6S RNA, and M1 RNA at RNA sizes of 76 nt, 120 nt, 183 nt, and 377 nt, respectively, and can be used for RNA size measurement of other small RNAs.
