# Supplementary material for: Combined Transcriptomic and Proteomic Profiling of E. coli under Microaerobic versus Aerobic Conditions: The Multifaceted Roles of Noncoding Small RNAs and Oxygen-Dependent Sensing in Global Gene Expression Control
Source: Int J Mol Sci. 2022 Feb 25;23(5):2570. doi: 10.3390/ijms23052570 (PMC8910356; doi:10.3390/ijms23052570)
Supplement: Supplementary file 1 [file ijms-23-02570-s001.zip › ijms-1593126-revised-supplement-20220217-01.pdf]

International Journal of
Molecular Sciences

MDPI

Combined transcriptomic and proteomic profiling of *E. coli* under microaerobic versus aerobic conditions: the multifaceted roles of non-coding small RNAs and oxygen-dependent sensing in global gene expression control

Gunn-Guang Liou[1], Anna Chao Kaberdina*[1], Wei-Syuan Wang*[1,2], Vladimir R. Kaberdin[3,4,5] and Sue Lin-Chao**[1,2]**

[1]Institute of Molecular Biology, Academia Sinica, Taipei, Taiwan
[2]Molecular and Cell Biology, Taiwan International Graduate Program, Institute of Molecular Biology, Academia Sinica and Graduate Institute of Life Sciences, National Defense Medical Center, Taipei, Taiwan
[3]Department of Immunology, Microbiology and Parasitology, University of the Basque Country UPV/EHU, 48940 Leioa, Spain
[4]IKERBASQUE, Basque Foundation for Science, Maria Diaz de Haro 3, 48013 Bilbao, Spain
[5]Research Centre for Experimental Marine Biology and Biotechnology (PIE-UPV/EHU), 48620 Plentzia, Spain
*AC Kaberdina and W-S Wang contributed equally to this work
** Correspondence author: mbsue@gate.sinica.edu (S.L - C); vladimir.kaberdin@ehu.eus (V.R.K.)
Authorship contributions:
GGL, ACK & WSW: Design, perform and analysis of the work.
GGL, VRK & SLC: Design, Interpretation of the data, drafting and revising the work.
SLC: Supervise the work.

Figure legends

Figure S1. Transcriptome data quality assessment. (A) Individual correlation coefficients (r) of pair-wise scatterplots are shown. The specific r values for aerobic (samples O-1~O-5) and micro-aerobic (samples N-1~N-10) growth conditions are presented in tables at top-right and bottom-left, respectively. (B) Principal component analysis (PCA) illustrates the relationships among all test samples, which form two distinct clusters. The green and blue dots represent the datasets obtained for RNA isolated from the microaerobic (N-1~N-10) and aerobic cultures (O-1~O-5), respectively.

Figure S2. Proteomic data quality assessment. Pair-wise scatter plots for aerobically grown samples (O-1 and O-2, left panel) and microaerobically grown samples (N-2 and N-3, right panel). Correlation coefficients (R^2) are 0.98 and 0.97 for the samples grown under aerobic and microaerobic conditions, respectively.
